# On coding genotypes for genetic markers with multiple alleles in genetic association study of quantitative traits

**DOI:** 10.1186/1471-2156-12-82

**Published:** 2011-09-21

**Authors:** Tao Wang

**Affiliations:** 1 Division of Biostatistics, Institute for Health and Society, Medical College of Wisconsin, Milwaukee, WI 53226, USA

## Abstract

**Background:**

In genetic association study of quantitative traits using F_∞ _models, how to code the marker genotypes and interpret the model parameters appropriately is important for constructing hypothesis tests and making statistical inferences. Currently, the coding of marker genotypes in building F_∞ _models has mainly focused on the biallelic case. A thorough work on the coding of marker genotypes and interpretation of model parameters for F_∞ _models is needed especially for genetic markers with multiple alleles.

**Results:**

In this study, we will formulate F_∞ _genetic models under various regression model frameworks and introduce three genotype coding schemes for genetic markers with multiple alleles. Starting from an allele-based modeling strategy, we first describe a regression framework to model the expected genotypic values at given markers. Then, as extension from the biallelic case, we introduce three coding schemes for constructing fully parameterized one-locus F_∞ _models and discuss the relationships between the model parameters and the expected genotypic values. Next, under a simplified modeling framework for the expected genotypic values, we consider several reduced one-locus F_∞ _models from the three coding schemes on the estimability and interpretation of their model parameters. Finally, we explore some extensions of the one-locus F_∞ _models to two loci. Several fully parameterized as well as reduced two-locus F_∞ _models are addressed.

**Conclusions:**

The genotype coding schemes provide different ways to construct F_∞ _models for association testing of multi-allele genetic markers with quantitative traits. Which coding scheme should be applied depends on how convenient it can provide the statistical inferences on the parameters of our research interests. Based on these F_∞ _models, the standard regression model fitting tools can be used to estimate and test for various genetic effects through statistical contrasts with the adjustment for environmental factors.

## Background

Genetic markers with multiple alleles are common phenomena in genetic studies. It is well known that the ABO blood types in human are determined by three alleles at a genetic locus on chromosome 9. Molecular markers such as microsatellites often have multiple alleles. The major histocompatibility complex (MHC), a highly polymorphic genome region that resides on the human chromosome 6, encompasses multiple genes that encode for many human leukocyte antigens (HLA) and play an important role in regulation of the immune responses. Depending on the resolution level of allele typing, each of the HLA-A, B, C, DR, DQ and DP gene loci could contain tens to hundreds of allele types. In addition, in the haplotype analysis of single-nucleotide polymorphisms (SNPs), various haplotypes from a set of SNPs can also be treated as different alleles from a 'super' marker locus that consists of the set of SNPs.

Presently, there are mainly three types of genetic models that are commonly used in the genetic analysis of quantitative traits. One is Fisher's analysis of variance (ANOVA) models that focus on a decomposition of the genotypic variance into genetic variance components contributed by various genetic effects at quantitative trait loci (QTL) [1-6]. Another is the F_∞ _models that concentrate on direct statistical modeling of the expected genotypic values at target genetic markers or QTL and the association testing of various genetic effects. The other one is the so-called functional genetic models that emphasize on modeling the functional effects of genes [7]. Both Fisher's and F_∞ _models can be referred to as statistical models, while the functional genetic models have fundamentally different objectives and estimation methods from the statistical models. A considerable amount of discussion has been made about the distinction between these different types of genetic models [8-11].

The F_∞ _models have been widely used in genetic association studies of quantitative traits. In building F_∞ _models, how to code genotypes at a marker (or QTL) and interpret the model parameters are fundamental issues for constructing appropriate testing hypotheses and making correct statistical inferences. While the Fisher's ANOVA models can be directly applicable to genetic markers with multiple alleles, the F_∞ _models by contrast have been mainly discussed in the biallelic case [1,9,12]. For haplotype analysis, Zaykin *et al*. in [13] proposed a simple coding which included only the additive effects of haplotypes but ignored their interactions. More recently, Yang *et al*. in [11] explored an extension of the biallelic F_∞ _models to multi-allele models with a focus on the definition of various genetic effects and their relationships with the average genetic effects defined in the Fisher's models. A thorough work on coding of marker genotypes and interpretation of model parameters for F_∞ _models has not been done in the past especially for genetic markers with multiple alleles.

In general, there are two different strategies in coding the marker or QTL genotypes. One is to treat each marker or QTL as a potential risk factor with its genotypes as the risk units. Then, similar to the strategy in handling categorical covariates in classical regression models, at each locus we can create one dummy variable per genotype and then include all but one (as the reference) of these dummy variables into a model. But this genotype coding is often limited by the available sample sizes especially when the number of alleles at the marker locus is large. Alternatively, as alleles are often supposed to be the basic genetic risk units that may contribute to disease phenotypes in genetic studies, we may want to treat alleles at each marker or QTL as the risk units and examine the effects of alleles. However, genetic data has some specialty that needs to be taken into account in order to build the allele-based models. In the genome of diploid species such as human being, alleles normally appear in pairs to form a genotype at each marker locus or QTL with one from the father and one from the mother, except for the sex chromosomes in males. That is, at each locus we have two within-locus risk factors that reside on a homologous pair of chromosomes. Unlike the classical two-way ANOVA model in which the two risk factors own different risk units, the paternal and maternal risk factors at a locus often share the same set of alleles. Besides, the parental origins (i.e., the phase) of the two alleles at each locus are quite often unknown. These features could sometimes complicate the allele-based coding of marker genotypes and generate confusion in interpretation of the model parameters.

In this study, we introduce three allele-based coding schemes for building F_∞ _models, namely allele, F_∞ _and allele-count codings. First, we formulate F_∞ _models under a general regression framework to model the expected genotypic values at given markers or QTL. Then, under a standard ANOVA model setting, we present several fully parameterized one-locus models using the three allele-based coding schemes. Some potential collinearity relationships among the coding variables of the marker genotypes are clarified. Strategies to avoid the redundant model parameters are also proposed. After that, we examine the definition of model parameters under a reduced one-locus model framework. The impact of a linear relationship among the coding variables of marker genotypes on the estimability of the model parameters is fully explored based on the linear model theory. Finally, we consider extension of the one-locus models to two-locus situation. Several fully parameterized as well as reduced two-locus models are addressed. A focus of this study is to establish the relationships between the model parameters and the expected genotypic values at given marker loci or QTL for various F_∞ _models from these three coding schemes under various different model frameworks, and explain how to estimate and test for various genetic effects through statistical contrasts. Relationships among different coding schemes and models are also illustrated through simulation.

## Results

### Fully parameterized one-locus models

In genetic studies, a quantitative trait *Y *is typically considered as a combination of a genetic component *G *and an environmental component *E *with perhaps the genetic by environmental interactions *G *× *E*, where *G *is the true genotypic value from a joint (unobservable) contribution of all the genetic factors to the quantitative trait *Y*. In practice, given a random sample of *N *individuals from a study population, let *g_i _*be the observed genotypes at certain target marker loci or QTL and *z_i _*be a vector of some environmental covariates that may contribute to the variation of the quantitative trait for individuals *i *= 1, ..., *N*. By ignoring the genetic by environmental interactions and assuming that the genotypic value *G *and environmental component *E *do not depend on the environmental covariates *z_i _*and *g_i_*, respectively, then the observed quantitative trait *y_i _*of an individual *i *can be expressed through a regression model as

(1) $$y_{i}=G\left(g_{i}\right)+z_{i}\beta +e_{i},i=1,\ldots ,N$$

where *G*(*g_i_*) = E(*G*|*g_i_*) is the expected genotypic value of *G *given the marker (or QTL) genotypes *g_i_*, *β *denotes the effects of the environmental covariates, and *e_i _*is the residual error of the model with E(*e_i_*) = 0. Similar to introducing dummy variables for the covariates *z_i _*which allow us to assess various environmental effects *β *in the model, it is convenient to further represent *G*(*g_i_*) as *G*(*g_i_*) = *x*(*g_i_*)*α *so that we can fit the regression model and assess the genetic effects *α *of the markers or QTL, where *x*(*g_i_*) is a coding function of the marker genotypes. When the marker locus is not associated with the phenotype, then *G*(*g_i_*) = E(*G*) is a constant which does not depend on *g_i_*. In the rest of the paper, we will focus on the interpretation of the marker effects *α *in terms of the expected genotypic values *G*(*g*) = E(*G*|*g*) according to different coding schemes. When certain genetic by environmental interactions are included in the model, the interpretation of *α *could be modified accordingly. It has to be pointed out that QTL are generally assumed to be unknown genomic regions that may contribute to the variation of the quantitative traits with their genotypes unobserved. But the results (i.e., the coding schemes and the relationships between the model parameters and the expected genotypic values) are held for QTL as well, although the expected genotypic values at a target QTL can no longer be directly estimated via fitting the regression models.

Now, consider one target marker locus with multiple alleles *A*_1_, ..., *A_m_*, *m *≥ 2. In general, there are *m *possible homozygous genotypes *A_j_A_j_*, *j *= 1 ..., *m*, and *m*(*m *- 1)/2 possible heterozygous genotypes *A_j_A_k_*, *j *≠ *k*. Let *G_jk _*= E(*G*|*g *= *A_j_A_k_*) be the expected genotypic values, given the marker genotypes *A_j_A_k _*in a study population. Without knowing the parental origins of the alleles, we assume as usual that the parental origin of the alleles does not make a difference (i.e., no imprinting). We have then *G_jk _*= *G_kj _*for *j*, *k *= 1, ..., *m*, and there are totally *m*(*m *+ 1)/2 possible distinctive expected genotypic values *G_jk_*, *j*, *k *= 1, ..., *m*, which could be estimated through the means in the genotypic subgroups after adjustment for the environmental covariates. Here we assume no missing genotypes for the sampled individuals, and the random sample has its individuals carrying all possible genotypes. How to handle missing genotypes will be discussed in the discussion. To fully re-parameterize these expected genotypic values through a linear model, we then need totally *m*(*m *+ 1)/2 parameters including the intercept in the model. By treating the paternal and maternal alleles as two independent risk factors and following the classical two-way ANOVA notation, we can represent the genotypic values *G_jk _*as

(2) $$G_{jk}=\mu^{*}+\alpha_{j}^{*}+\alpha_{k}^{*}+\delta_{jk}^{*},j,k=1,\ldots ,m$$

where $\alpha_{j}^{*}$ and $\delta_{jk}^{*}$ are the realized (but unobservable) additive effects of allele *A_j _*and the allelic interaction between the two alleles *A_j _*and *A_k_*, respectively. The above model is different from the classical two-way ANOVA model in that here both the paternal and the maternal risk factors share the same set of alleles *A*_1_, ..., *A_m_*. As usual, with the unknown paternal origins of alleles at the locus, we assume the paternal and maternal alleles have the same genetic effect. More precisely, the paternal allele *A_j _*and maternal allele *A_j _*have the same additive allelic effects $\alpha_{j}^{*}$ for *j *= 1, ..., *m*. Besides, the allelic interaction between a paternal allele *A_j _*and a maternal allele *A_k _*is the same as that between the paternal allele *A_k _*and the maternal allele *A_j_*; i.e., $\delta_{jk}^{*}=\delta_{kj}^{*}$, for *j*, *k *= 1, ..., *m*. Still, with *m *additive allelic effects and *m*(*m *+ 1)/2 allelic interactions plus the intercept, it is clear that model (2) is over-parameterized on modeling the *m*(*m *+ 1)/2 expected genotypic values *G_jk _*for *j*, *k *= 1, ..., *m*. As a result, the parameters μ*, $\alpha_{j}^{*}$ and $\delta_{jk}^{*}$ in model (2) are not all estimable in terms of the expected genotypic values *G_jk _*(see [14,15]).

In order to avoid the inestimability issue, one way is to add constraints on the model parameters. However, those constraints, together with the symmetry property of $\delta_{jk}^{*}$, could make it difficult to fit the model using the standard software package such as SAS. Alternatively, we consider dropping certain redundant parameters in the model. Similar to the biallelic case [10], let us first introduce the following indicator variables to describe the transmission of alleles from parents to their offspring

$$z_{1j}=\left\{\begin{array}{c} 1,\;\text{inherited}\;A_{j}\;\text{on}\;\text{paternal}\;\text{gamete},\\ 0,\;\text{inherited}\;\text{other}\;\text{alleles}\;\text{on}\;\text{paternal}\;\text{gamete} \end{array}\right)$$

and

$$z_{2j}=\left\{\begin{array}{c} 1,\;\text{inherited}\;A_{j}\;\text{on}\;\text{maternal}\;\text{gamete},\\ 0,\;\text{inherited}\;\text{other}\;\text{alleles}\;\text{on}\;\text{maternal}\;\text{gamete} \end{array}\right)$$

for each allele type *A_j_*, *j *= 1, ..., *m*. Then we define the following coding variables of the marker genotypes

$$\begin{array}{c} w_{j}\left(g\right)=z_{1j}+z_{2j}=\left\{\begin{array}{c} 2,\;\text{if}\;g=A_{j}A_{j}\\ 1,\;\text{if}\;g=A_{j}A_{j}^{c}\\ 0,\;\text{if}\;g=A_{j}^{c}A_{j}^{c} \end{array}\right)\\ v_{jk}\left(g\right)=z_{1j}z_{2k}=\left\{\begin{array}{c} 1,\;\text{if}\;g=A_{j}A_{k}\\ 0,\;\text{otherwise} \end{array}\right) \end{array}$$

for *j*, *k *= 1, ..., *m*, where $A_{j}^{c}$ denotes any other allele type except *A_j_*. Note that *z*_1*j*_, *z*_2*j*_are not observable because we do not know exactly which allele is inherited from paternal or maternal gamete for the sampled individuals without their parental information. But this unknown phase problem does not affect the definitions of *w_j_*, *v_jk _*since *w_j _*only counts the number of allele *A_j _*in the genotypes and the value of *v_jk _*is 1 when the genotype is *A_j_A_k _*and 0 otherwise regardless of where the two alleles come from. We refer to the above coding of marker genotypes as an allele coding scheme. Model (2) can then be re-written in a linear model form as

(3) $$G\left(g_{i}\right)=\mu^{*}+\overset{m}{\underset{j=1}{\sum }}\alpha_{j}^{*}w_{j}\left(g_{i}\right)+\overset{m}{\underset{j=1}{\sum }}\overset{m}{\underset{k=j}{\sum }}\delta_{jk}^{*}v_{jk}\left(g_{i}\right)$$

for *i *= 1, ..., *N*. As each individual always carries two alleles at a marker locus with one from the father and the other from the mother, we have $\sum_{j=1}^{m}z_{1j}\left(g_{i}\right)=\sum_{k=1}^{m}z_{2k}\left(g_{i}\right)=1$, for any *i *= 1, ..., *N*. Therefore, given a particular *j*, $w_{jk}=2-\sum_{k\neq j}w_{k}$, which is a linear combination of the rest of {*w_k_*, *k *≠ *j*}. For *v_jk_*, we also have $\sum_{j=1}^{m}v_{jk}=z_{2k}$, or $v_{jk}=w_{k}/2-\sum_{l\neq j}v_{lk}$. Hence, each of the *v_jk_*, *k *= 1, ..., *m*, is also a linear combination of the coding variables {*w_k_*, *k *≠ *j*} and {*v_lk_*, *l*, *k *≠ *j*}. To avoid the redundancy of parameters due to these collinearity relationships among the coding variables in model (3), without losing generality, we consider dropping *w_m _*and {*v_km_*, *k *= 1, ..., *m*} in (3). Then

(4) $$G\left(g_{i}\right)=\mu +\overset{m-1}{\underset{j=1}{\sum }}\alpha_{j}w_{j}\left(g_{i}\right)+\overset{m-1}{\underset{j=1}{\sum }}\overset{m-1}{\underset{k=j}{\sum }}\delta_{jk}v_{jk}\left(g_{i}\right)$$

for *i *= 1, ..., *N*. Model (4) now provides a full re-parameterization of the *m*(*m *+ 1)/2 expected genotypic values *G_jk _*for *j*, *k *= 1, ..., *m *with its parameters *α_j _*can be referred to as the additive allelic effects and *δ_jk _*the allelic interactions with respect to the reference allele *A_m_*. Given a random sample, we can then incorporate model (4) into (1) and fit the regression model (1) using the standard least-square approach. In terms of the expected genotypic values, it is easy to show that *μ *= *G_mm_*, *α_j _*= *G_jm _*- *G_mm _*and *δ_jk _*= (*G_jk _*- *G_km_*) - (*G_jm _*- *G_mm_*), for *j *= 1, ..., *m *- 1 and *k *= *j*, ..., *m *- 1. Therefore, the additive allelic effect *α_j _*can be interpreted as the substitution effect of replacing allele *A_m _*by *A_j _*when paired with another allele *A_m _*to form the genotypes. Meanwhile, the allelic interaction *δ_jk _*is the difference between the substitution effect of replacing allele *A_m _*by *A_j _*(or *A_k_*) when paired with allele *A_k _*(or *A_j_*) and that when paired with allele *A_m_*. Or, in other words, *δ_jk _*is the difference between the substitution effects of replacing allele *A_m _*by *A_j _*(or *A_k_*) with paired alleles *A_k _*(or *A_j_*) and *A_m_*. Note that dropping *w_j _*and {*v_kj_*, *k *= 1, ..., *m*} for a particular *j *≠ *m *instead of *w_m _*and {*v_km_*, *k *= 1, ..., *m*} can lead to similar interpretations of the model parameters with *A_j _*being the reference allele. Using model (4), we can also estimate and test for various other genetic effects. For example, the so-called functional 'additive effects' $a_{jk}^{*}=\left(G_{jj}-G_{kk}\right)∕2$ and the 'dominance effects' $d_{jk}^{*}=G_{jk}-\left(G_{jj}+G_{kk}\right)∕2$, *j *≠ *k *defined in [11] can be expressed as $a_{jk}^{*}=\left(\alpha_{j}-\alpha_{k}\right)+\left(\delta_{jj}-\delta_{kk}\right)∕2$ and $d_{jk}^{*}=\delta_{jk}-\left(\delta_{jj}+\delta_{kk}\right)∕2-2\mu$, *j *≠ *k*, respectively, in terms of the above model parameters. So we can estimate $a_{jk}^{*}$, $d_{jk}^{*}$ using the fitted model parameters or test for the hypothesis of $H_{0}:a_{jk}^{*}=0$ or $H_{0}:d_{jk}^{*}=0$ through the general linear contrasts [15] using the standard software such as SAS. To test whether a particular allele *A_j _*has an overall effect, the null hypothesis is *H*_0 _: *α_j _*= *δ_jk _*= 0 for *k *= 1, ⋯, *m *- 1, which can be performed through either a general linear contrast (or likelihood ratio test) with the degrees of freedom being *m *for the test statistic. The association test for overall effects of the locus corresponds to the null hypothesis of *H*_0 _: *α_j _*= *δ_jk _*= 0 for any *j*, *k *= 1, ⋯, *m *- 1, which has its degrees of freedom being *m*(*m *+ 1)/2 - 1 for the test statistic. Currently, the so-called F_∞ _model has been widely used in genetic association studies. In the simple biallelic case with two alleles *A *and *α*, an F_∞ _model gives [16-19].

$$G_{AA}=\tau +a,\;G_{Aa}=\tau +d,\;G_{aa}=\tau -a$$

where *G_AA _*= E(*G*|*AA*), *G_Aa _*= E(*G*|*Aa*) and *G_aa _*= E(*G*|*aa*) are the three possible expected genotypic values at the marker. The parameters *a*, *d *are often referred to as the additive and dominance effects of the allele *A *over *a*, and in terms of the expected genotypic values we have *a *= (*G_AA _*- *G_aa_*)/2 and *d *= *G_Aa _*- (*G_AA _*+ *G_aa_*)/2. This F_∞ _model can also be written in a linear model form as [10]

$$G\left(g_{i}\right)=\tau +af\left(g_{i}\right)+dh\left(g_{i}\right),i=1,\ldots ,N$$

where *f*, *h *are two coding variables of the marker genotypes that are defined as

$$\begin{array}{c} f\left(g\right)=\left\{\begin{array}{cc} 1, & \text{if}\;g=AA\\ 0, & \text{if}\;g=Aa\\ -1, & \text{if}\;g=aa \end{array}\right)\\ h\left(g\right)=\left\{\begin{array}{cc} 1, & \text{if}\;g=Aa\\ 0, & \text{otherwise} \end{array}\right) \end{array}$$

We refer to the above coding of the marker genotypes as the F_∞ _coding. As a straightforward extension of the F_∞ _coding scheme to multiple alleles, we can define the following coding variables

$$\begin{array}{c} f_{j}\left(g\right)=\left\{\begin{array}{cc} 1, & if\;g=A_{j}A_{j}\\ 0, & \text{if}\;g=A_{j}A_{j}^{c}\\ -1, & \text{if}\;g=A_{j}^{c}A_{j}^{c} \end{array}\right)\\ h_{j}\left(g\right)=\left\{\begin{array}{cc} 1, & \text{if}\;g=A_{j}A_{j}^{c}\\ 0, & \text{otherwise} \end{array}\right) \end{array}$$

for each *j *= 1, ..., *m*. It is easy to see that *f_j_*, *h_j _*and the previous *w_j_*, *v_jk_*, *j*, *k *= 1, ..., *m *have the relationships: *f_j_*(*g*) = *w_j_*(*g*) - 1, *h_j_*(*g*) = *w_j_*(*g*) - 2*v_jj_*(*g*), and *v_jk_*(*g*) = *h_j_*(*g*)*h_k_*(*g*) as *j *≠ *k*. Thus, for the same reason to avoid collinearity, we can exclude some redundant coding variables and write a fully parameterized one-locus model using the F_∞ _coding as

(5) $$\begin{array}{c} G\left(g_{i}\right)=\tau +\overset{m-1}{\underset{j=1}{\sum }}a_{j}f_{j}\left(g_{i}\right)+\overset{m-1}{\underset{j=1}{\sum }}d_{jj}h_{j}\left(g_{i}\right)\\ \;+\overset{m-1}{\underset{j=1}{\sum }}\overset{m-1}{\underset{k=j+1}{\sum }}d_{jk}h_{j}\left(g_{i}\right)h_{k}\left(g_{i}\right) \end{array}$$

for *i *= 1, ..., *N*. By having model (5) equivalent to (4), we can first build the relationships between the two model parameters and then establish the relationships between the parameters of model (5) and the expected genotypic values as following

$$\left\{\begin{array}{c} \tau =\mu +\overset{m}{\underset{j=1}{\sum }}\left(\alpha_{j}+\frac{\delta_{jj}}{2}\right)\\ \;=G_{mm}+\frac{1}{2}\sum_{j=1}^{m-1}\left(G_{jj}-G_{mm}\right)\\ a_{j}=\alpha_{j}+\frac{\delta_{jj}}{2}=\frac{G_{jj}-G_{mm}}{2},j=1,\ldots ,m-1\\ d_{jj}=-\frac{\delta_{jj}}{2}=G_{jm}-\frac{G_{jj}+G_{mm}}{2},j=1,\ldots ,m-1\\ d_{jk}=\delta_{jk}=\left(G_{jk}-G_{jm}\right)-\left(G_{km}-G_{mm}\right),j\neq k \end{array}\right)$$

Therefore, *a_j _*can be interpreted as a half of the difference between the two expected homozygous genotypic values *G_jj _*and *G_mm_*, which is the same as the additive effect $a_{jm}^{*}$ defined in [11]. Besides, *d_jj _*is the difference between the expected heterozygous genotypic value *G_jm _*and the averaged expected homozygous genotypic value (*G_jj _*+ *G_mm_*)/2, which is the same as the dominance effect $d_{jm}^{*}$ defined in [11]. It is interesting to see that *d_jk_*, *j *≠ *k*, has the same interpretation as *δ_jk _*in model (4), which is the difference between the substitution effects of replacing allele *A_m _*by *A_j _*when paired with alleles *A_k _*and *A_m_*. Note that *d_jj _*can also be interpreted as the allelic interaction - the difference between the substitution effects of replacing allele *A_j _*by *A_m _*when paired with another *A_j _*and *A_m_*. In addition, based on model (5), the additive effects $a_{jk}^{*}$ and the dominance effects $d_{jk}^{*}$ proposed in [11] have the relationship with the model parameters: $a_{jk}^{*}=a_{j}-a_{k}$, $d_{jk}^{*}=d_{jk}+\left(d_{jj}+d_{kk}\right)$, *j *≠ *k*. The overall effect of a particular allele *A_j _*can be tested through the composite hypothesis of *H*_0 _: *a_j _*= *d_jk _*= 0 for *k *= 1, ⋯, *m *- 1, and the overall effects of the locus can be tested via the null hypothesis of *H*_0 _: *a_j _*= *d_jk _*= 0 for any *j*, *k *= 1, ⋯, *m *- 1.

In addition to the allele and F_∞ _codings, another way of coding the marker genotypes which occasionally appears in practice is to count the number of alleles in marker genotypes for each specific allele *A_j_*. As each individual can have 0, 1 or 2 copies of an allele *A_j_*, by taking the genotypic group with 0 copy of allele *A_j _*as the baseline, we can introduce the following two indicator (or dummy) variables for the genotypic groups with 1 and 2 copies of the allele *A_j_*, respectively.

$$\begin{array}{c} h_{1j}\left(g\right)=\left\{\begin{array}{c} 1,\;\text{if}\;g=A_{j}A_{j}^{c}\\ 0,\;\text{otherwise} \end{array}\right)\\ h_{2j}\left(g\right)=\left\{\begin{array}{c} 1,\;\text{if}\;g=A_{j}A_{j}\\ 0,\;\text{otherwise} \end{array}\right) \end{array}$$

for each *j *= 1, ..., *m *- 1. These coding variables of marker genotypes have relationships *h*_1*j*_(*g*) = *h_j_*(*g*) = *w_j_*(*g*) - 2*v_jj_*(*g*) and *h*_2*j*_(*g*) = *v_jj_*(*g*) with previous ones. We refer to this coding of marker genotypes as the allele-count coding. Similar to models (4) and (5), by excluding some redundant coding variables, the allele-count coding leads to another fully parameterized one-locus model as

(6) $$\begin{array}{c} G\left(g_{i}\right)=\pi_{0}+\overset{m-1}{\underset{j=1}{\sum }}\pi_{j}h_{1j}\left(g_{i}\right)+\overset{m-1}{\underset{j=1}{\sum }}\eta_{jj}h_{2j}\left(g_{i}\right)\\ \;+\overset{m-1}{\underset{j=1}{\sum }}\overset{m-1}{\underset{k=j+1}{\sum }}\eta_{jk}h_{1j}\left(g_{i}\right)h_{1k}\left(g_{i}\right) \end{array}$$

for *i *= 1, ..., *N*. Similarly, by having model (6) equivalent to (4), we can establish the following relationships

$$\left\{\begin{array}{c} \pi_{0}=\mu =G_{mm}\\ \pi_{j}=\alpha_{j}=G_{jm}-G_{mm},j=1,\ldots ,m-1\\ \eta_{jj}=2\alpha_{j}+\delta_{jj}=G_{jj}-G_{mm},j=1,\ldots ,m-1\\ \eta_{jk}=\delta_{jk}=\left(G_{jk}-G_{jm}\right)-\left(G_{km}-G_{mm}\right),j\neq k \end{array}\right)$$

Therefore, *π_j _*in model (6) can still be interpreted as the substitution effect of replacing allele *A_m _*by *A_j _*when paired with allele *A_m_*, or the difference between the genotypic values of the genotype group *A_j_A_m _*with one copy of *A_j _*versus the genotype group *A_m_A_m _*(baseline). *η_jj _*is the difference between the expected genotypic value *G_jj _*in the homozygous genotypic group *A_j_A_j _*with two copies of *A_j _*and *G_mm _*in the baseline group *A_m_A_m_*. Besides, *η_jk _*in model (6) has the same interpretation as *δ_jk _*(or *d_jk_*) before. From model (6), the general additive effects $a_{jk}^{*}=\left(\eta_{jj}-\eta_{kk}\right)∕2$ and the dominance effects $d_{jk}^{*}=\eta_{jk}-\left(\eta_{jj}+\eta_{kk}\right)∕2-2\pi_{0}$, *j *≠ *k*, which can be tested either separately or jointly. The overall effect of a particular allele *A_j _*can be tested through the composite hypothesis of *H*_0 _: *π_j _*= *η_jk _*= 0 for *k *= 1, ⋯, *m *- 1. The overall effects of the locus can also be tested via the null hypothesis of *H*_0 _: *π_j _*= *η_jk _*= 0 for any *j*, *k *= 1, ⋯, *m *- 1.

Each of the three models (4), (5) and (6) provides a full re-parameterization of the *m*(*m *+ 1)/2 expected genotypic values under the same model framework (3). The relationships between their model parameters and the expected genotypic values are summarized in Table 1. It is interesting to see from Table 1 that the null hypothesis of *α_j _*= *δ_jj _*= 0 is equivalent to either *a_j _*= *d_jj _*= 0 or *π_j _*= *η_jj _*= 0, which implies *G_jj _*= *G_jm _*= *G_mm_*. So the three models above should provide the same test statistics for testing *α_j _*= *δ_jj _*= 0, *a_j _*= *d_jj _*= 0 or *π_j _*= *η_jj _*= 0.

**Table 1:** Parameterization of fully parameterized one-locus models (4), (5), (6).

Codings	Relationships
Allele	$\begin{array}{c} \mu =G_{mm},\alpha_{j}=G_{jm}-G_{mm}\\ \delta_{jj}=G_{jj}+G_{mm}-2G_{jm},j=1,\ldots ,m-1\\ \delta_{jk}=\left(G_{jk}-G_{jm}\right)-\left(G_{km}-G_{mm}\right),j,k=1,\ldots ,m-1;j<k \end{array}$

F_∞_	$\begin{array}{c} \tau =G_{mm}+\frac{1}{2}\sum_{j=1}^{m-1}\left(G_{jj}-G_{mm}\right)\\ a_{j}=\frac{G_{jj}-G_{mm}}{2},d_{jj}=G_{jm}-\frac{G_{jj}+G_{mm}}{2},j=1,\ldots ,m-1\\ d_{jk}=\left(G_{jk}-G_{jm}\right)-\left(G_{km}-G_{mm}\right),j,k=1,\ldots ,m-1;j<k \end{array}$

Allele-count	$\begin{array}{c} \pi_{0}=G_{mm},\pi_{j}=G_{jm}-G_{mm}\\ \eta_{jj}=G_{jj}-G_{mm},j=1,\ldots ,m-1\\ \eta_{jk}=\left(G_{jk}-G_{jm}\right)-\left(G_{km}-G_{mm}\right),j,k=1,\ldots ,m-1;j<k \end{array}$

For a biallelic locus with alleles *A *(or *A*_1_) and *a *(or *A*_2_), we have *m *= 2 with three possible genotypic values *G_AA _*= *E*(*G*|*AA*), *G_Aa _*= *E*(*G*|*Aa*) and *G_aa _*= *E*(*G*|*aa*). If we adopt the allele coding, then *w*_2_(*g*) = 2 - *w*_1_(*g*), *v*_12_(*g*) = *w*_1_(*g*) - *v*_11_(*g*), and *v*_22_(*g*) = 1 - *w*_1_(*g*) + *v*_11_(*g*). For the F_∞ _coding, we have *f*_2_(*g*) = -*f*_1_(*g*) and *h*_2_(*g*) = *h*_1_(*g*). So we can further drop *d*_2 _in model (5). For the allele-count coding, we have *h*_12_(*g*) = *h*_11_(*g*) and *h*_22_(*g*) = 1 - *h*_11_(*g*) - *h*_21_(*g*). The interpretation of model parameters for these three biallelic QTL models are summarized in Table 2, which is a special case of Table 1.

**Table 2:** Parameterization of one-locus models (4), (5), (6) when *m *= 2.

Codings	Models	Relationships
Allele	$\begin{array}{c} G_{AA}=\mu +2\alpha_{1}+\delta_{11}\\ G_{Aa}=\mu +\alpha_{1}\\ G_{aa}=\mu \end{array}$	$\begin{array}{c} \mu =G_{aa}\\ \alpha_{1}=G_{Aa}-G_{aa}\\ \delta_{11}=G_{AA}+G_{aa}-2G_{Aa} \end{array}$

F_∞_	$\begin{array}{c} G_{AA}=\tau +a_{1}\\ G_{Aa}=\tau +d_{11}\\ G_{aa}=\tau -a_{1} \end{array}$	$\begin{array}{c} \tau =\frac{G_{AA}+G_{aa}}{2}\\ a_{1}=\frac{G_{AA}-G_{aa}}{2}\\ d_{11}=G_{Aa}-\frac{G_{AA}+G_{aa}}{2} \end{array}$

Allele-count	$\begin{array}{c} G_{AA}=\pi_{0}+\eta_{11}\\ G_{Aa}=\pi_{0}+\pi_{1}\\ G_{aa}=\pi_{0} \end{array}$	$\begin{array}{c} \pi_{0}=G_{aa}\\ \pi_{1}=G_{Aa}-G_{aa}\\ \eta_{11}=G_{AA}-G_{aa} \end{array}$

For a locus with three alleles *A*_1_, *A*_2 _(i.e., *m *= 3), we have six possibly distinctive expected genotypic values *G*_11_, *G*_22_, *G*_33_, *G*_12_, *G*_13 _and *G*_23_. Each of the three fully parameterized models (4), (5) and (6) can provide a full re-parameterization of the six expected genotypic values. In a matrix form, from the allele coding model (4), we have

$$\left[\begin{array}{c} G_{11}\\ G_{22}\\ G_{33}\\ G_{12}\\ G_{13}\\ G_{23} \end{array}\right]=\left[\begin{array}{cccccc} 1 & 2 & 0 & 1 & 0 & 0\\ 1 & 0 & 2 & 0 & 1 & 0\\ 1 & 0 & 0 & 0 & 0 & 0\\ 1 & 1 & 1 & 0 & 0 & 1\\ 1 & 1 & 0 & 0 & 0 & 0\\ 1 & 0 & 1 & 0 & 0 & 0 \end{array}\right]\left[\begin{array}{c} \mu \\ \alpha_{1}\\ \alpha_{2}\\ \delta_{11}\\ \delta_{22}\\ \delta_{12} \end{array}\right]$$

From the F_∞ _coding model (5), we have

$$\left[\begin{array}{c} G_{11}\\ G_{22}\\ G_{33}\\ G_{12}\\ G_{13}\\ G_{23} \end{array}\right]=\left[\begin{array}{cccccc} 1 & 1 & -1 & 0 & 0 & 0\\ 1 & -1 & 1 & 0 & 0 & 0\\ 1 & -1 & -1 & 0 & 0 & 0\\ 1 & 0 & 0 & 1 & 1 & 1\\ 1 & 0 & -1 & 1 & 0 & 0\\ 1 & -1 & 0 & 0 & 1 & 0 \end{array}\right]\left[\begin{array}{c} \tau \\ a_{1}\\ a_{2}\\ d_{11}\\ d_{22}\\ d_{12} \end{array}\right]$$

And the allele-count coding model (6) gives

$$\left[\begin{array}{c} G_{11}\\ G_{22}\\ G_{33}\\ G_{12}\\ G_{13}\\ G_{23} \end{array}\right]=\left[\begin{array}{cccccc} 1 & 0 & 0 & 1 & 0 & 0\\ 1 & 0 & 0 & 0 & 1 & 0\\ 1 & 0 & 0 & 0 & 0 & 0\\ 1 & 1 & 1 & 0 & 0 & 1\\ 1 & 1 & 0 & 0 & 0 & 0\\ 1 & 0 & 1 & 0 & 0 & 0 \end{array}\right]\left[\begin{array}{c} \pi_{0}\\ \pi_{1}\\ \pi_{2}\\ \eta_{11}\\ \eta_{22}\\ \eta_{12} \end{array}\right]$$

By multiplying the design matrices on the left side of the equations, we can show that the model parameters and the expected genotypic values have the relationships as summarized in Table 3, which is consistent with that in Table 1.

**Table 3:** Parameterization of one-locus models (4), (5), (6) when *m *= 3.

Codings	Relationships
Allele	$\begin{array}{c} \mu =G_{33}\\ \alpha_{1}=G_{13}-G_{33},\alpha_{2}=G_{23}-G_{33}\\ \delta_{11}=G_{11}+G_{33}-2G_{13}\\ \delta_{22}=G_{22}+G_{33}-2G_{23}\\ \delta_{12}=G_{12}+G_{33}-G_{13}-G_{23} \end{array}$

F_∞_	$\begin{array}{c} \tau =\frac{G_{11}+G_{22}}{2}\\ a_{1}=\frac{G_{11}-G_{33}}{2},a_{2}=\frac{G_{22}-G_{33}}{2}\\ d_{11}=G_{13}-\frac{G_{11}+G_{33}}{2}\\ d_{22}=G_{23}-\frac{G_{22}+G_{33}}{2}\\ d_{12}=G_{12}+G_{33}-G_{13}-G_{23} \end{array}$

Allele-count	$\begin{array}{c} \pi_{0}=G_{33}\\ \pi_{1}=G_{13}-G_{33},\pi_{2}=G_{23}-G_{33}\\ \eta_{11}=G_{11}-G_{33}\\ \eta_{22}=G_{22}-G_{33}\\ \eta_{12}=G_{12}+G_{33}-G_{13}-G_{23} \end{array}$

### Reduced one-locus models

Due to limited available sample sizes in practice, it may not always be feasible to use the fully parameterized models. Quite often, one may want to check the main effects of alleles first before including all possible allelic interactions. Here we consider the case of including possible interactions between *A_j _*and itself for the homozygous genotypes *A_j _A_j_*, *j *= 1, ..., *m*, but ignore other interactions between different alleles *A_j _*and *A_k _*(*j *≠ *k*). Then we obtain a reduced case of model (2) as below

(7) $$G_{jk}=\mu^{*}+\alpha_{j}^{*}+\alpha_{k}^{*}+\delta_{j}^{*}1_{\left\{j=k\right\}}$$

for *j*, *k *= 1, ..., *m*. Similarly, using the allele coding, we can present this model in a linear model form as

(8) $$G\left(g_{i}\right)=\mu^{*}+\overset{m}{\underset{j=1}{\sum }}\alpha_{j}^{*}w_{j}\left(g_{i}\right)+\overset{m}{\underset{j=1}{\sum }}\delta_{j}^{*}v_{j}\left(g_{i}\right)$$

for *i *= 1, ..., *N*, where *v_j_*(*g*) = *v_jj_*(*g*) for *j *= 1, ..., *m*, with *v_jj_*(*g*) defined as before.

Model (8) contains only one redundant parameter in the α*'s due to the fact that $\sum_{j=1}^{m}w_{j}\left(g_{i}\right)=2$ for *i *= 1, ..., *N*. In this case, as shown in Appendix A, the parameters $\delta_{1}^{*},\;.\;.\;.\;,\delta_{m}^{*}$ in model (8) are estimable but the parameters μ* and $\alpha_{1}^{*},\;.\;.\;.\;,\alpha_{m}^{*}$ are not estimable. To overcome the redundant parameter problem, we can drop *w_m _*from model (8) and consider

(9) $$G\left(g_{i}\right)=\mu +\overset{m-1}{\underset{j=1}{\sum }}\alpha_{j}w_{j}\left(g_{i}\right)+\overset{m}{\underset{j=1}{\sum }}\delta_{j}v_{j}\left(g_{i}\right)$$

for *i *= 1, ..., *N*. Note that $v_{m}=z_{1m}z_{2m}=1-\sum_{j=1}^{m-1}w_{j}+\sum_{j=1}^{m-1}\sum_{k=1}^{m-1}v_{jk}$, which cannot be completely determined by {*w_j_*, *v_j_*, *j *= 1, ..., *m *- 1}. Therefore, dropping {*δ_jk_*, *j*, *k *= 1, ..., *m *- 1, *j *<*k*} from model (4) does not directly lead to an equivalent model of (9) as the latter contains *v_m_*. In fact, as further dropping *v_m _*in (9), it will lead to a more restricted model structure for the expected genotypic values with the similar interpretation of its model parameters as presented in model (4). It is also interesting to see that the haplotype coding proposed in [13] is a special case of model (9) when we further ignore all the allelic interactions and drop all the {*v_j_*, *j *= 1, ..., *m*} in the model.

By definition, a reduced model can be derived from its original model by adding certain restrictions on the model parameters. Typically, the model parameters in a reduced model could be interpreted similarly as that in its original model when these restrictions are simple enough (e.g., by setting a subset of them being zero). When the restrictions on the original model parameters are complicated, however, the interpretation of the reduced model parameters could be different from that presented in the original model. For model (9), we can establish the relationship between its model parameters and the expected genotypic values using a classical matrix approach, as shown in Appendix B. An alternative way of building this relationship is to simply treat model (9) as a reduced form of model (8) by adding a restriction $\alpha_{m}^{*}=0$ and taking *μ *= μ*, $\alpha_{j}=\alpha_{j}^{*}$ for *j *= 1, ..., *m *- 1, and $\delta_{j}=\delta_{j}^{*}$ for *j *= 1, ..., *m*. Note that adding the restriction $\alpha_{m}^{*}=0$ on (8) does not change the modeling structure of the expected genotypic values because $\alpha_{m}^{*}$ is a redundant parameter given the others. Therefore,

$$\left\{\begin{array}{c} \mu =G_{mm}-\delta_{m}^{*}=G_{jm}+G_{km}-G_{jk},\\ \;\;j\neq k\neq m\\ \alpha_{j}=G_{jm}-\mu^{*}=G_{jk}-G_{km},\;k\neq j,m,\\ \;\;j=1,\ldots ,m-1\\ \delta_{j}=G_{jj}-\left(\mu^{*}+2\alpha_{j}^{*}\right)\\ \;=\left(G_{jj}-G_{jk}\right)-\left(G_{jl}-G_{kl}\right),j\neq k\neq l,\\ \;\;j=1,\ldots ,m \end{array}\right)$$

Comparing with the parameters in model (4), we can see that the interpretation of the parameters in model (9) have changed slightly. The intercept *μ *now becomes $\left(G_{mm}-\delta_{m}^{*}\right)$ instead of *G_mm_*, the *α_j _*is the substitution effect of replacing allele *A_m _*by *A_j _*when paired with any allele *A_k _*(*k *≠ *j*, *m*) instead of just *A_m_*, while the *δ_j _*is the difference between the substitution effect of replacing any allele *A_k _*by *A_j _*when paired with *A_j _*itself and that when paired with another allele *A_l _*(*l *≠ *j*, *k*). If both *α_j _*and *δ_j _*are zero for a particular *j *<*m*, then *G_jj _*= *G_jm _*= *μ *and *G_jk _*= *G_km _*for any *k *≠ *j*, *m*.

Under the same model framework (8), the F_∞ _coding leads to the following model

(10) $$G\left(g_{i}\right)=\tau +\overset{m-1}{\underset{j=1}{\sum }}a_{j}f_{j}\left(g_{i}\right)+\overset{m}{\underset{j=1}{\sum }}d_{j}h_{j}\left(g_{i}\right)$$

for *i *= 1, ..., *N*. By applying the relationship *f_j_*(*g*) = *w_j_*(*g*) - 1 and *h_j_*(*g*) = *w_j_*(*g*) - 2*v_j_*(*g*) for *j *= 1, ..., *m*, we can show that for models (10) and (8) to be equivalent their model parameters have the relationship

$$\left\{\begin{array}{c} \tau =\mu^{*}+\overset{m}{\underset{j=1}{\sum }}\left(\alpha_{j}^{*}+\frac{\delta_{j}^{*}}{2}\right)\\ a_{j}=\alpha_{j}^{*}+\frac{\delta_{j}^{*}}{2},j=1,\ldots ,m-1\\ d_{j}=-\frac{\delta_{j}^{*}}{2},j=1,\ldots ,m\\ \alpha_{m}^{*}+\frac{\delta_{m}^{*}}{2}=0 \end{array}\right)$$

In other words, model (10) leads to a restriction $2\alpha_{m}^{*}+\delta_{m}^{*}=0$ on the parameters in model (8) which makes $\mu^{*}=G_{mm}-\left(2\alpha_{m}^{*}+\delta_{m}^{*}\right)=G_{mm}$, $\alpha_{m}^{*}=-\delta_{m}^{*}/2$ and $\alpha_{j}^{*}=G_{jm}-\left(\mu^{*}+\alpha_{m}^{*}\right)=G_{jm}-G_{mm}+\delta_{m}^{*}/2$, *j *= 1, ..., *m *- 1. Thus,

$$\left\{\begin{array}{c} \tau =G_{mm}+\frac{1}{2}\overset{m-1}{\underset{j=1}{\sum }}\left(G_{jj}-G_{mm}\right)\\ a_{j}=\frac{G_{jj}-G_{mm}}{2},j=1,\ldots ,m-1\\ d_{j}=-\frac{\left(G_{jj}-G_{jk}\right)-\left(G_{jl}-G_{kl}\right)}{2},j\neq k\neq l,\\ \;\;j=1,\ldots ,m \end{array}\right)$$

Now *d_j _*becomes a half of the difference between the substitution effect of replacing any allele *A_k _*by *A_j _*when paired with another *A_j _*and that when paired with an allele *A_l _*(*l *≠ *j*, *k*), which can no longer be referred to as a dominance effect.

With the allele-count coding, we can actually construct two equivalent models in this case

(11) $$G\left(g_{i}\right)=\pi_{0}+\overset{m-1}{\underset{j=1}{\sum }}\pi_{j}h_{1j}\left(g_{i}\right)+\overset{m}{\underset{j=1}{\sum }}\eta_{j}h_{2j}\left(g_{i}\right)$$

and

(12) $$G\left(g_{i}\right)=\pi_{0}^{\prime }+\overset{m}{\underset{j=1}{\sum }}{\pi^{\prime }}_{j}h_{1j}\left(g_{i}\right)+\overset{m-1}{\underset{j=1}{\sum }}{\eta^{\prime }}_{j}h_{2j}\left(g_{i}\right)$$

for *i *= 1, ..., *N*. Similarly, we can show that model (11) can be treated as a reduced model by adding the restriction $\alpha_{m}^{*}=0$ on parameters in model (8) with the following relationships

$$\left\{\begin{array}{c} \pi_{0}=\mu^{*}=G_{jm}+G_{km}-G_{jk},\\ \;\;\;j\neq k\neq m\\ \pi_{j}=\alpha_{j}^{*}=G_{jk}-G_{km},k\neq j,m,\\ \;\;\;j=1,\ldots ,m-1\\ \eta_{j}=2\alpha_{j}^{*}+\delta_{j}^{*}=\left(G_{jj}-G_{jm}\right)+\left(G_{jk}-G_{km}\right),\\ \;\;\;k\neq j,m,j=1,\ldots ,m-1\\ \eta_{m}=\delta_{m}^{*}=\left(G_{mm}-G_{jm}\right)-\left(G_{km}-G_{jk}\right),\\ \;\;\;j\neq k\neq m \end{array}\right)$$

On the other hand, model (12) can be treated as a reduced model by adding the restriction $2\alpha_{m}^{*}+\delta^{*}=0$ on parameters in model (10) with the following relationships

$$\left\{\begin{array}{c} {\pi^{\prime }}_{0}=\mu^{*}=G_{mm}\\ {\pi^{\prime }}_{j}=\alpha_{j}^{*}=\frac{\left(G_{jm}-G_{mm}\right)+\left(G_{jk}-G_{km}\right)}{2},\\ \;\;\;k\neq j,m,j=1,\ldots ,m-1\\ {\pi^{\prime }}_{m}=-\frac{\delta_{m}^{*}}{2}=-\frac{\left(G_{mm}-G_{jm}\right)-\left(G_{km}-G_{jk}\right)}{2},\\ \;\;\;j\neq k\neq m\\ {\eta^{\prime }}_{j}=2\alpha_{j}^{*}+\delta_{j}^{*}=G_{jj}-G_{mm},\\ \;\;\;j=1,\ldots ,m-1 \end{array}\right)$$

While the effect *η_jj _*in model (6) is the difference between the two expected homozygous genotypic values *G_jj _*and *G_mm_*, the effect *η_j _*in model (11) becomes the sum of the substitution effects of replacing allele *A_m _*by *A_j _*when paired with *A_j _*itself and when paired with another allele *A_k _*(*k *≠ *j*, *m*. It is also interesting to see that the definition of parameters in models (11) and (12) are quite different. A null hypothesis of $H_{0}:\pi_{j}^{\prime }=\eta_{j}^{\prime }=0$ for a particular *j *<*m *in model (12) implies that *G_jj _*= *G_mm _*and *G_jm _*- *G_mm _*= *G_jk _*- *G_km _*for any *k *≠ *j*, *m*, while the null hypothesis of *H*_0 _: *π_j _*= *η_j _*= 0 for a *j *<*m *in model (11) implies that *G_jj _*= *G_jm _*and *G_jk _*= *G_km _*for any *k *≠ *j*, *m*, which has nothing to do with *G_mm_*.

Under the same model framework (8), each of the above four models (9), (10), (11) and (12) contains 2*m *non-redundant parameters (including the intercept) to model the *m*(*m *+ 1)/2 expected genotypic values. When *m *> 3, we have *m*(*m *+ 1)/2 > 2*m*. Therefore, the model framework (7) enforces certain constraints on the *m*(*m *+ 1)/2 genotypic values. If *m *= 3, then each of the four models actually provides a full re-parameterization of the six expected genotypic values *G*_11_, *G*_22_, *G*_33_, *G*_12_, *G*_13 _and *G*_23_. The relationships between the four model parameters and the expected genotypic values are summarized in Table 4.

**Table 4:** Parameterization of one-locus models (9), (10), (11), (12) when *m *≥ 3.

Codings	Restrictions	Relationships
Allele	$\alpha_{m}^{*}=0$	$\begin{array}{c} \mu =\mu^{*}=\left(G_{jm}+G_{km}\right)-G_{jk},j\neq k\neq m\\ \alpha_{j}=\alpha_{j}^{*}=G_{jk}-G_{km},j=1,\ldots ,m-1;j\neq k,m\\ \delta_{j}=\delta_{j}^{*}=\left(G_{jj}-G_{jk}\right)-\left(G_{jl}-G_{kl}\right),j=1,\ldots ,m;k\neq j\neq l \end{array}$

F_∞_	$2\alpha_{m}^{*}+\delta_{m}^{*}=0$	$\begin{array}{c} \tau =\mu^{*}+\frac{1}{2}\overset{m-1}{\underset{j=1}{\sum }}\left(2\alpha_{j}^{*}+\delta_{j}^{*}\right)=G_{mm}+\frac{1}{2}\overset{m-1}{\underset{j=1}{\sum }}\left(G_{jj}-G_{mm}\right)\\ a_{j}=\frac{1}{2}\left(2\alpha_{j}^{*}+\delta_{j}^{*}\right)=\frac{G_{jj}-G_{mm}}{2},j=1,\ldots ,m-1\\ d_{j}=-\frac{\delta_{j}^{*}}{2}=-\frac{\left(G_{jj}-G_{jk}\right)-\left(G_{jl}-G_{kl}\right)}{2},j=1,\ldots ,m;j\neq k\neq l \end{array}$

Allele-count	$\alpha_{m}^{*}=0$	$\begin{array}{c} \pi_{0}=\mu^{*}=\left(G_{jm}+G_{km}\right)-G_{jk},j\neq k\neq m\\ \pi_{j}=\alpha_{j}^{*}=G_{jk}-G_{km},j=1,\ldots ,m-1;k\neq j,m\\ \eta_{j}=2\alpha_{j}^{*}+\delta_{j}^{*}=\left(G_{jj}-G_{jm}\right)+\left(G_{jk}-G_{km}\right),j=1,\ldots ,m-1;k\neq j,m\\ \eta_{m}=\delta_{m}^{*}=\left(G_{mm}-G_{jm}\right)-\left(G_{km}-G_{jk}\right),j\neq k\neq m \end{array}$

Allele-count	$2\alpha_{m}^{*}+\delta_{m}^{*}=0$	$\begin{array}{c} {\pi^{\prime }}_{0}=\mu^{*}=G_{mm}\\ {\pi^{\prime }}_{j}=\alpha_{j}^{*}=\frac{\left(G_{jm}-G_{mm}\right)+\left(G_{jk}-G_{km}\right)}{2},j=1,\ldots ,m-1;k\neq j,m\\ {\pi^{\prime }}_{m}=-\frac{\delta_{m}^{*}}{2}=-\frac{\left(G_{mm}-G_{jm}\right)-\left(G_{km}-G_{jk}\right)}{2},j\neq k\neq m\\ {\eta^{\prime }}_{j}=2\alpha_{j}^{*}+\delta_{j}^{*}=G_{jj}-G_{mm},j=1,\ldots ,m-1 \end{array}$

Comparing Table 4 with Table 1, we can see that the definition of model parameters depends not only on the coding schemes of marker genotypes but also on the underlying framework for the structure of the expected genotypic values. From Table 4, it is also interesting to see that the null hypothesis of *H*_0 _: *α_j _*= *δ_j _*= 0 (*j *<*m*) in model (9) is equivalent to *π_j _*= *η_j _*= 0 in model (11), which implies $\alpha_{j}^{*}=\delta_{j}^{*}=0$ in model (8) with restriction $\alpha_{m}^{*}=0$, or *G_jk _*= *G_km _*for any *k *= 1, ..., *m*. On the other hand, the null hypothesis of *H*_0 _: *a_j _*= *d_j _*= 0 (*j *<*m*) in model (10) is equivalent to $\pi_{j}^{\prime }=\eta_{j}^{\prime }=0$ in model (12), which implies $\alpha_{j}^{*}=\delta_{j}^{*}=0$ in model (8) with a restriction $2\alpha_{m}^{*}+\delta_{m}^{*}=0$, or *G_jj _*= *G_mm _*and *G_jj _*- *G_jm _*= *G_jk _*- *G_km _*for any *k *≠ *m*. In general, the two null hypotheses of *α_j _*= *δ_j _*= 0 and *a_j _*= *d_j _*= 0 may not always be equivalent. For example, when *m *= 3, similar to the three-allele models discussed in the previous section, we can show that the four model parameters and the expected genotypic values have the relationships as shown in Table 5, which is a special case of Table 4. We can see from Table 5 that *α*_1 _= *δ*_1 _= 0 is equivalent to *π*_1 _= *η*_1 _= 0 which implies *G*_12 _= *G*_23 _and *G*_11 _= *G*_13_; while *a*_1 _= *d*_1 _= 0 is equivalent to $\pi_{1}^{\prime }=\eta_{1}^{\prime }=0$ which implies *G*_11 _= *G*_33 _and *G*_12 _+ *G*_13 _= *G*_11 _+ *G*_23_. So, depending on the underlying true setting of the expected genotypic values, the null hypotheses of *α*_1 _= *δ*_1 _= 0 in model (9) could be different from that of *a*_1 _= *d*_1 _= 0 in model (10).

**Table 5:** Parameterization of one-locus models (9), (10), (11), (12) when *m *= 3.

Codings	Restrictions	Relationships
Allele	$\alpha_{3}^{*}=0$	$\begin{array}{c} \mu =G_{13}+G_{23}-G_{12}\\ \alpha_{1}=G_{12}-G_{23},\alpha_{2}=G_{12}-G_{13}\\ \delta_{1}=\left(G_{11}-G_{13}\right)-\left(G_{12}-G_{23}\right)\\ \delta_{2}=\left(G_{22}-G_{23}\right)-\left(G_{12}-G_{13}\right)\\ \delta_{3}=G_{33}+G_{12}-G_{13}-G_{23} \end{array}$

F_∞_	$2\alpha_{3}^{*}+\delta_{3}^{*}=0$	$\begin{array}{c} \tau =\frac{G_{11}+G_{22}}{2}\\ a_{1}=\frac{G_{11}-G_{33}}{2},a_{2}=\frac{G_{22}-G_{33}}{2}\\ d_{1}=\frac{\left(G_{12}+G_{13}\right)-\left(G_{23}+G_{11}\right)}{2}\\ d_{2}=\frac{\left(G_{12}+G_{23}\right)-\left(G_{13}+G_{22}\right)}{2}\\ d_{3}=\frac{\left(G_{13}+G_{23}\right)-\left(G_{12}+G_{33}\right)}{2} \end{array}$

Allele-count	$\alpha_{3}^{*}=0$	$\begin{array}{c} \pi_{0}=G_{13}+G_{23}-G_{12}\\ \pi_{1}=G_{12}-G_{23},\pi_{2}=G_{12}-G_{13}\\ \eta_{1}=\left(G_{11}-G_{13}\right)+\left(G_{12}-G_{23}\right)\\ \eta_{2}=\left(G_{22}-G_{23}\right)+\left(G_{12}-G_{13}\right)\\ \eta_{3}=G_{33}+G_{12}-G_{13}-G_{23} \end{array}$

Allele-count	$2\alpha_{3}^{*}+\delta_{3}^{*}=0$	$\begin{array}{c} \pi_{0}^{\prime }=G_{33}\\ \pi_{1}^{\prime }=\frac{\left(G_{12}+G_{13}\right)-\left(G_{23}+G_{33}\right)}{2}\\ \pi_{2}^{\prime }=\frac{\left(G_{12}+G_{23}\right)-\left(G_{13}+G_{33}\right)}{2}\\ \pi_{3}^{\prime }=\frac{\left(G_{13}+G_{23}\right)-\left(G_{12}+G_{33}\right)}{2}\\ \eta_{1}^{\prime }=G_{11}-G_{33},\;\eta_{2}^{\prime }=G_{22}-G_{33} \end{array}$

### Extension to two-locus models

In this section, we further explore some extensions of the previous one-locus models to two-locus models. Consider two marker loci with alleles $A_{11},\ldots ,A_{1m_{1}}$ at locus 1 and alleles $A_{21},\ldots ,A_{2m_{2}}$ at locus 2, respectively. Without distinguishing the parental origins of the alleles, there are totally *m*_1_*m*_2_(*m*_1 _+ 1)(*m*_2 _+ 1)/4 possible distinctive expected genotypic values: *G_jkrs _*= E(*G*|*A*_1*j*_*A*_1*k*_*A*_2*r*_*A*_2*s*_) for *j*, *k *= 1, ..., *m*_1_, *j *≤ *k*; and *r*, *s *= 1, ..., *m*_2_, *r *≤ *s*. Using the allele coding, we introduce the following coding variables

$$\begin{array}{c} w_{1j}\left(g\right)=\left\{\begin{array}{cc} 2, & \text{if}\;g=A_{1j}A_{1j}\\ 1, & \text{if}\;g=A_{1j}A_{1j}^{c}\\ 0, & \text{if}\;g=A_{1j}^{c}A_{1j}^{c} \end{array}\right)\\ v_{1jk}\left(g\right)=\left\{\begin{array}{cc} 1, & \text{if}\;g=A_{1j}A_{1k}\\ 0, & \text{otherwise} \end{array}\right) \end{array}$$

*j*, *k *= 1, ..., *m*_1_, for marker genotypes at locus 1 and

$$w_{2r}\left(g\right)=\left\{\begin{array}{cc} 2, & \text{if}\;g=A_{2r}A_{2r}\\ 1, & \text{if}\;g=A_{2r}A_{2r}^{c}\\ 0, & \text{if}\;g=A_{2r}^{c}A_{2r}^{c} \end{array}\right)$$

$$v_{2rs}\left(g\right)=\left\{\begin{array}{cc} 1, & \text{if}\;g=A_{2r}A_{2s}\\ 0, & \text{otherwise} \end{array}\right)$$

*r*, *s *= 1, ..., *m*_2_, for marker genotypes at locus 2, where $A_{1j}^{c}$ (or $A_{2r}^{c}$) denotes any other allele type except *A*_1*j*_(or *A*_2*r*_) at locus 1 (or 2). A fully parameterized two-locus model for *G_jkrs _*can then be presented as

(13) $$\begin{array}{c} G\left(g_{i}\right)=\mu +\overset{m_{1}-1}{\underset{j=1}{\sum }}\alpha_{1j}w_{1j}+\overset{m_{1}-1}{\underset{j=1}{\sum }}\overset{m_{1}-1}{\underset{k=j}{\sum }}\delta_{1jk}v_{1jk}\\ +\overset{m_{2}-1}{\underset{r=1}{\sum }}\alpha_{2r}w_{2r}+\overset{m_{2}-1}{\underset{r=1}{\sum }}\overset{m_{2}-1}{\underset{s=r}{\sum }}\delta_{2rs}v_{2rs}\\ +\overset{m_{1}-1}{\underset{j=1}{\sum }}\overset{m_{2}-1}{\underset{r=1}{\sum }}\left(\alpha_{1j}\alpha_{2r}\right)w_{1j}w_{2r}\\ +\overset{m_{1}-1}{\underset{j=1}{\sum }}\overset{m_{2}-1}{\underset{r=1}{\sum }}\overset{m_{2}-1}{\underset{s=r}{\sum }}\left(\alpha_{1j}\delta_{2rs}\right)w_{1j}v_{2rs}\\ +\overset{m_{1}-1}{\underset{j=1}{\sum }}\overset{m_{1}-1}{\underset{k=j}{\sum }}\overset{m_{2}-1}{\underset{r=1}{\sum }}\left(\delta_{1jk}\alpha_{2r}\right)v_{1jk}w_{2r}\\ +\overset{m_{1}-1}{\underset{j=1}{\sum }}\overset{m_{1}-1}{\underset{k=j}{\sum }}\overset{m_{2}-1}{\underset{r=1}{\sum }}\overset{m_{2}-1}{\underset{s=r}{\sum }}\left(\delta_{1jk}\delta_{2rs}\right)v_{1jk}v_{2rs} \end{array}$$

for *i *= 1, ..., *N*. Similar to the one-locus models, we can establish the relationship between the model parameters and the expected genotypic values as shown in (C.1) of Appendix C. A nice property of this allele coding model is that a higher order effect is simply the deviation of its corresponding expected genotypic value from an approximation of the other lower order effects. Here the corresponding expected genotypic value of a marker effect is determined by the position of alleles that differ from the two reference alleles $A_{1m_{1}}$ and $A_{2m_{2}}$. So, starting from the lowest order parameter *μ*, it seems straightforward to build the relationships between the model parameters and the expected genotypic values starting from the low-order effect parameters up to the high-order effect parameters.

For the F_∞ _coding, we can define the following coding variables for the genotypes at the two marker loci separately.

$$\begin{array}{c} f_{1j}\left(g\right)=\left\{\begin{array}{cc} 1, & if\;g=A_{1j}A_{1j}\\ 0, & if\;g=A_{1j}A_{1j}^{c}\\ -1, & if\;g=A_{1j}^{c}A_{1j}^{c} \end{array}\right)\\ h_{1j}\left(g\right)=\left\{\begin{array}{cc} 1, & if\;g=A_{1j}A_{1j}^{c}\\ 0, & \text{otherwise} \end{array}\right) \end{array}$$

for *j *= 1, ..., *m*_1_, and

$$\begin{array}{c} f_{2r}\left(g\right)=\left\{\begin{array}{cc} 1, & if\;g=A_{2r}A_{2r}\\ 0, & if\;g=A_{2r}A_{2r}^{c}\\ -1, & if\;g=A_{2r}^{c}A_{2r}^{c} \end{array}\right)\\ h_{2r}\left(g\right)=\left\{\begin{array}{cc} 1, & if\;g=A_{2r}A_{2r}\\ 0, & \text{otherwise} \end{array}\right) \end{array}$$

for *r *= 1, ..., *m*_2_. A fully parameterized two-locus model using this F_∞ _coding is then

(14) $$\begin{array}{c} G\left(g_{i}\right)=\tau +\overset{m_{1}-1}{\underset{j=1}{\sum }}a_{1j}f_{1j}\left(g_{i}\right)+\overset{m_{2}-1}{\underset{r=1}{\sum }}a_{2r}f_{2r}\left(g_{i}\right)\\ \;+\overset{m_{1}-1}{\underset{j=1}{\sum }}\overset{m_{1}-1}{\underset{k=j}{\sum }}d_{1jk}h_{1j}\left(g_{i}\right)h_{1k}\left(g_{i}\right)\\ \;+\overset{m_{2}-1}{\underset{r=1}{\sum }}\overset{m_{2}-1}{\underset{s=r}{\sum }}d_{2rs}h_{2r}\left(g_{i}\right)h_{2s}\left(g_{i}\right)\\ \;+\overset{m_{1}-1}{\underset{j=1}{\sum }}\overset{m_{2}-1}{\underset{r=1}{\sum }}\left(a_{1j}a_{2r}\right)f_{1j}f_{2r}\\ \;+\overset{m_{1}-1}{\underset{j=1}{\sum }}\overset{m_{2}-1}{\underset{r=1}{\sum }}\overset{m_{2}-1}{\underset{s=r}{\sum }}\left(a_{1j}d_{2rs}\right)f_{1j}h_{2r}h_{2s}\\ \;+\overset{m_{1}-1}{\underset{j=1}{\sum }}\overset{m_{1}-1}{\underset{k=j}{\sum }}\overset{m_{2}-1}{\underset{r=1}{\sum }}\left(d_{1jk}a_{2r}\right)h_{1j}h_{1k}f_{2r}\\ \;+\overset{m_{1}-1}{\underset{j=1}{\sum }}\overset{m_{1}-1}{\underset{k=j}{\sum }}\overset{m_{2}-1}{\underset{r=1}{\sum }}\overset{m_{2}-1}{\underset{s=r}{\sum }}\left(d_{1jk}d_{2rs}\right)h_{1j}h_{1k}h_{2r}h_{2s} \end{array}$$

for *i *= 1, ..., *N*. Still, using the relationships *w*_1*j*_= 1 + *f*_1*j*_, *w*_2*r*_= 1 + *f*_2*r*_, *v*_1*jj*_= (1 + *f*_1*j*_- *h*_1*j*_), *v*_2*rr*_= (1 + *f*_2*r*_- *h*_2*r*_), *v*_1*jk*_= *h*_1*j*_*h*_1*k*_for *j *≠ *k*, and *v*_2*rs*_= *h*_2*r*_*h*_2*s*_for *r *≠ *s *between the F_∞ _coding variables and the allele coding variables, we can establish the relationships between the model parameters and the expected genotypic values as shown in (C.2) of Appendix C. We can easily verify that the biallelic two-locus effects $E_{F_{\infty }\cdot AB}$ in [9] is a special case of our results with *m*_1 _= *m*_2 _= 2. It is also interesting to see that the interpretation of model parameters in terms of the expected genotypic values becomes much more complicated than that in the previous allele coding model. When *m*_1_, *m*_2 _> 2, the low-order within-locus main effect *a*_1*j*_is a weighted combination of the differences $\left(G_{jjrr}-G_{m_{1}m_{1}rr}\right)$, where *r *= 1, ..., *m*_2 _refer to various homozygous genotypes *A*_2*r*_*A*_2*r*_at locus 2. The within-locus effect *d*_1*jj*_is a weighted combination of the allelic interactions $\left(G_{jjrr}-2G_{jm_{1}rr}+G_{m_{1}m_{1}rr}\right)$, *r *= 1, ..., *m*_2_, at locus 1 with reference *A*_2*r*_*A*_2*r*_at locus 2. Even the intercept τ of the model becomes a complex function of various homozygous genotypic values.

Applying the allele-count coding, we can define

$$\begin{array}{c} h_{1j}^{\left(1\right)}\left(g\right)=\left\{\begin{array}{c} 1,if\;g=A_{1j}A_{1j}^{c}\\ 0,otherwise \end{array}\right)\\ h_{2j}^{\left(1\right)}\left(g\right)=\left\{\begin{array}{c} 1,if\;g=A_{1j}A_{1j}\\ 0,\text{otherwise} \end{array}\right) \end{array}$$

for *j *= 1, ..., *m*_1_, and

$$\begin{array}{c} h_{1r}^{\left(2\right)}\left(g\right)=\left\{\begin{array}{c} 1,if\;g=A_{2r}A_{2r}^{c}\\ 0,\text{otherwise} \end{array}\right)\\ h_{2r}^{\left(2\right)}\left(g\right)=\left\{\begin{array}{c} 1,if\;g=A_{2r}A_{2r}\\ 0,\text{otherwise} \end{array}\right) \end{array}$$

for *r *= 1, ..., *m*_2_. Another fully parameterized two-locus model for *G_jkrs _*can be written as

(15) $$\begin{array}{l} G(g_{i})=\pi_{0}+\sum_{j=1}^{m_{1}-1}\left(\pi_{1j}h_{1j}^{\left(1\right)}+\eta_{1jj}h_{2j}^{\left(1\right)}\right)\\ \;+\sum_{r=1}^{m_{2}-1}\left(\pi_{2r}h_{1r}^{\left(2\right)}+\eta_{2rr}h_{2r}^{\left(2\right)}\right)\\ \;+\sum_{j=1}^{m_{1}-1}\sum_{k=j+1}^{m_{1}-1}\eta_{1jk}h_{1j}^{\left(1\right)}h_{1k}^{\left(1\right)}\\ \;+\sum_{r=1}^{m_{2}-1}\sum_{s=r+1}^{m_{2}-1}\eta_{2rs}h_{1r}^{\left(2\right)}h_{1s}^{\left(2\right)}\\ \;+\sum_{j=1}^{m_{1}-1}\sum_{r=1}^{m_{2}-1}[(\pi_{1j}\pi_{2r})h_{1j}^{\left(1\right)}h_{1r}^{\left(2\right)}\\ \;\;+(\pi_{1j}\eta_{2rr})h_{1j}^{\left(1\right)}h_{2r}^{\left(2\right)}+(\eta_{1jj}\pi_{2r})h_{2j}^{\left(1\right)}h_{1r}^{\left(2\right)}\\ \;\;+(\eta_{1jj}\eta_{2rr})h_{2j}^{\left(1\right)}h_{2r}^{\left(2\right)}]\\ \;+\sum_{j=1}^{m_{1}-1}\sum_{r=1}^{m_{2}-1}\sum_{s=r+1}^{m_{2}-1}[(\pi_{1j}\eta_{2rs})h_{1j}^{\left(1\right)}h_{1r}^{\left(2\right)}h_{1s}^{\left(2\right)}\\ \;\;+(\eta_{1jj}\eta_{2rs})h_{2j}^{\left(1\right)}h_{1r}^{\left(2\right)}h_{1s}^{\left(2\right)}]\\ \;+\sum_{j=1}^{m_{1}-1}\sum_{k=j+1}^{m_{1}-1}\sum_{r=1}^{m_{2}-1}[(\eta_{1jk}\pi_{2r})h_{1j}^{\left(1\right)}h_{1k}^{\left(1\right)}h_{1r}^{\left(2\right)}\\ \;\;+(\eta_{1jk}\eta_{2rr})h_{1j}^{\left(1\right)}h_{1k}^{\left(1\right)}h_{2r}^{\left(2\right)}]\\ \;\;+\sum_{j=1}^{m_{1}-1}\sum_{k=j+1}^{m_{1}-1}\sum_{r=1}^{m_{2}-1}\sum_{s=r+1}^{m_{2}-1}\left(\eta_{1jk}\eta_{2rs}\right)\\ \;\;\cdot h_{1j}^{\left(1\right)}h_{1k}^{\left(1\right)}h_{1r}^{\left(2\right)}h_{1s}^{\left(2\right)} \end{array}$$

for *i *= 1, ..., *N*. In this case, the allele-count coding variables and the allele coding variables have the relationships $w_{1j}=h_{1j}^{\left(1\right)}+2h_{2j}^{\left(1\right)}$, $w_{2r}=h_{1r}^{\left(2\right)}+2h_{2r}^{\left(2\right)}$, $v_{1jj}=h_{2j}^{\left(1\right)}$, $v_{2rr}=h_{2r}^{\left(2\right)}$, $v_{1jk}=h_{1j}^{\left(1\right)}h_{1k}^{\left(1\right)}$ for *j *≠ *k*, and $v_{2rs}=h_{1r}^{\left(2\right)}h_{1s}^{\left(2\right)}$ for *r *≠ *s*. Through the equivalence of the two models (13) and (15), we can also construct relationships between the parameters in model (15) and the expected genotypic values as shown in (C.3) of Appendix C. We can see that the interpretation of parameters in the allele-count coding model (15) are as simple as that in the allele coding model (13) with the same intercept being $G_{m_{1}m_{1}m_{2}m_{2}}$. Besides, it seems that some parameters such as (*η*_1*jj*_*η*_2*rr*_), (*η*_1*jk*_*η*_2*rs*_) and (*η*_1*jk*_*η*_2*rr*_) have simpler relationships than the corresponding ones in the allele coding model (13).

Finally, let us consider some reduced cases of the two-locus models. By ignoring locus-by-locus interactions (i.e., epistases), we have the following simplified two-locus model framework

(16) $$G_{jkrs}=\mu^{*}+\alpha_{1j}^{*}+\alpha_{1k}^{*}+\delta_{1jk}^{*}+\alpha_{2r}^{*}+\alpha_{2s}^{*}+\delta_{2rs}^{*}$$

for *j*, *k *= 1, ..., *m*_1 _and *r*, *s *= 1, ..., *m*_2_. If we further ignore the within-locus allelic interactions between different alleles, then another reduced two-locus model framework is

(17) $$\begin{array}{c} G_{jkrs}=\mu^{*}+\alpha_{1j}^{*}+\alpha_{1k}^{*}+\delta_{1j}^{*}1_{\left\{j=k\right\}}\\ +\;\alpha_{2r}^{*}+\alpha_{2s}^{*}+\delta_{2r}^{*}1_{\left\{r=s\right\}} \end{array}$$

Similar to the one-locus models, under each of the two reduced model frameworks we can construct the two-locus models from the three coding schemes. The relationships between the model parameters and the expected genotypic values under framework (14) are summarized in Table 6, which can be treated as an extension of Table 1 to the two-locus case. The relationships between the model parameters and the expected genotypic values under framework (17) are also summarized in Table 7, which is a straightforward extension of Table 4. Further dropping $\delta_{1j}^{*}$ for *j *= 1, ..., *m*_1 _and $\delta_{2r}^{*}$ for *r *= 1, ..., *m*_2 _in (15) will lead to an additive model framework, which has its model parameters interpretable similar to that in Table 6. From Tables 6 and 7, we can see that both the allele and allele-count coding models have their lower-order main effects keep similar interpretation as to that in the previous fully parameterized case with epistases, while the F_∞ _coding models have the definition of their lower-order main effects vary depending on whether there are epistases involved in the models.

**Table 6:** Parameterization of two-locus models under model framework (16).

Codings	Relationships
Allele	$\begin{array}{c} \mu =G_{m_{1}m_{1}m_{2}m_{2}}\\ \alpha_{1j}=G_{jm_{1}m_{2}m_{2}}-G_{m_{1}m_{1}m_{2}m_{2}},j=1,\ldots ,m_{1}-1\\ \alpha_{2r}=G_{m_{1}m_{1}rm_{2}}-G_{m_{1}m_{1}m_{2}m_{2}},r=1,\ldots ,m_{2}-1\\ \delta_{1jk}=G_{jkm_{2}m_{2}}-G_{jm_{1}m_{2}m_{2}}-G_{km_{1}m_{2}m_{2}}+G_{m_{1}m_{1}m_{2}m_{2}},j,k=1,\ldots ,m_{1}-1;j\leq k\\ \delta_{2rs}=G_{m_{1}m_{1}rs}-G_{m_{1}m_{1}rm_{2}}-G_{m_{1}m_{1}sm_{2}}+G_{m_{1}m_{1}m_{2}m_{2}},r,s=1,\ldots ,m_{2}-1;r\leq s \end{array}$

F_∞_	$\begin{array}{c} \tau =G_{m_{1}m_{1}m_{2}m_{2}}+\frac{1}{2}\sum_{j=1}^{m_{1}-1}\left(G_{jjm_{2}m_{2}}-G_{m_{1}m_{1}m_{2}m_{2}}\right)+\frac{1}{2}\sum_{r=1}^{m_{2}-1}\left(G_{m_{1}m_{1}rr}-G_{m_{1}m_{1}m_{2}m_{2}}\right)\\ a_{1j}=\frac{G_{jjm_{2}m_{2}}-G_{m_{1}m_{1}m_{2}m_{2}}}{2},j=1,\ldots ,m_{1}-1\\ a_{2r}=\frac{G_{m_{1}m_{1}rr}-G_{m_{1}m_{1}m_{2}m_{2}}}{2},r=1,\ldots ,m_{2}-1\\ d_{1jj}=G_{jm_{1}m_{2}m_{2}}-\frac{G_{jjm_{2}m_{2}}+G_{m_{1}m_{1}m_{2}m_{2}}}{2},j=1,\ldots ,m_{1}-1\\ d_{1jk}=G_{jkm_{2}m_{2}}-G_{jm_{1}m_{2}m_{2}}-G_{km_{1}m_{2}m_{2}}+G_{m_{1}m_{1}m_{2}m_{2}},j,k=1,\ldots ,m_{1}-1;j<k\\ d_{2rr}=G_{m_{1}m_{1}rm_{2}}-\frac{G_{m_{1}m_{1}rr}+G_{m_{1}m_{1}m_{2}m_{2}}}{2},r=1,\ldots ,m_{2}-1\\ d_{2rs}=G_{m_{1}m_{1}rs}-G_{m_{1}m_{1}rm_{2}}-G_{m_{1}m_{1}sm_{2}}+G_{m_{1}m_{1}m_{2}m_{2}},r,s=1,\ldots ,m_{2}-1;r<s \end{array}$

Allele-count	$\begin{array}{c} \pi_{0}=G_{m_{1}m_{1}m_{2}m_{2}}\\ \pi_{1j}=G_{jm_{1}m_{2}m_{2}}-G_{m_{1}m_{1}m_{2}m_{2}},j=1,\ldots ,m_{1}-1\\ \pi_{2r}=G_{m_{1}m_{1}rm_{2}}-G_{m_{1}m_{1}m_{2}m_{2}},r=1,\ldots ,m_{2}-1\\ \eta_{1jj}=G_{jjm_{2}m_{2}}-G_{m_{1}m_{1}m_{2}m_{2}},j=1,\ldots ,m_{1}-1\\ \eta_{1jk}=G_{jkm_{2}m_{2}}-G_{jm_{1}m_{2}m_{2}}-G_{km_{1}m_{2}m_{2}}+G_{m_{1}m_{1}m_{2}m_{2}},j,k=1,\ldots ,m_{1}-1;j<k\\ \eta_{2rr}=G_{m_{1}m_{1}rr}-G_{m_{1}m_{1}m_{2}m_{2}},r=1,\ldots ,m_{2}-1\\ \eta_{2rs}=G_{m_{1}m_{1}rs}-G_{m_{1}m_{1}rm_{2}}-G_{m_{1}m_{1}sm_{2}}+G_{m_{1}m_{1}m_{2}m_{2}},r,s=1,\ldots ,m_{2}-1;r<s \end{array}$

**Table 7:** Parameterization of two-locus models under model framework (17) when *m*_1_, *m*_2 _≥ 3.

Codings	Restrictions	Relationships
Allele	$\alpha_{1m_{1}}^{*}=\alpha_{2m_{2}}^{*}=0$	$\begin{array}{c} \mu =G_{m_{1}m_{1}m_{2}m_{2}}-\left(G_{m_{1}m_{1}rs}-G_{jm_{1}rs}-G_{km_{1}rs}+G_{jkrs}\right)\\ \;-\left(G_{jkm_{2}m_{2}}-G_{jkm_{2}r}-G_{jkm_{2}s}+G_{jkrs}\right),j\neq k\neq m_{1};r\neq s\neq m_{2}\\ \alpha_{1j}=G_{jkrs}-G_{m_{1}krs},j=1,\ldots ,m_{1}-1;k\neq j,m_{1}\\ \alpha_{2r}=G_{jkrs}-G_{jkm_{2}s},r=1,\ldots ,m_{2}-1,r\neq s,m_{2}\\ \delta_{1j}=G_{jjrs}-G_{jkrs}-G_{jlrs}+G_{klrs},j=1,\ldots ,m_{1};j\neq k\neq l\\ \delta_{2r}=G_{jkrr}-G_{jkrs}-G_{jkrt}+G_{jkst},r=1,\ldots ,m_{2};r\neq s\neq t \end{array}$

F_∞_	$\begin{array}{c} 2\alpha_{1m_{1}}^{*}+\delta_{1m_{1}}^{*}=0\\ 2\alpha_{2m_{2}}^{*}+\delta_{2m_{2}}^{*}=0 \end{array}$	$\begin{array}{c} \tau =G_{m_{1}m_{1}m_{2}m_{2}}+\frac{1}{2}\sum_{j=1}^{m_{1}-1}\left(G_{jjm_{2}m_{2}}-G_{m_{1}m_{1}m_{2}m_{2}}\right)\\ \;+\frac{1}{2}\sum_{r=1}^{m_{2}-1}\left(G_{m_{1}m_{1}rr}-G_{m_{1}m_{1}m_{2}m_{2}}\right)\\ a_{1j}=\frac{G_{jjrs}-G_{m_{2}m_{2}rs}}{2},j=1,\ldots ,m_{1}-1\\ a_{2r}=\frac{G_{jkrr}-G_{jkm_{2}m_{2}}}{2},r=1,\ldots ,m_{2}-1\\ d_{1j}=-\frac{G_{jjrs}-G_{jkrs}-G_{jlrs}+G_{klrs}}{2},j=1,\ldots ,m_{1};j\neq k\neq l\\ d_{2r}=-\frac{G_{jkrr}-G_{jkrs}-G_{jkrt}+G_{jkst}}{2},r=1,\ldots ,m_{2};r\neq s\neq t \end{array}$

Allele-count	$\alpha_{1m_{1}}^{*}=\alpha_{2m_{2}}^{*}=0$	$\begin{array}{c} \pi_{0}=G_{m_{1}m_{1}m_{2}m_{2}}-\left(G_{m_{1}m_{1}rs}-G_{jm_{1}rs}-G_{m_{1}krs}+G_{jkrs}\right)\\ \;-\left(G_{jkm_{2}m_{2}}-G_{jkrm_{2}}-G_{jkm_{2}s}+G_{jkrs}\right),j\neq k\neq m_{1};r\neq s\neq m_{2}\\ \pi_{1j}=G_{jkrs}-G_{m_{1}krs},j=1,\ldots ,m_{1}-1,k\neq j,m_{1}\\ \pi_{2r}=G_{jkrs}-G_{jkm_{2}s},r=1,\ldots ,m_{2}-1,r\neq s,m_{2}\\ \eta_{1j}=G_{jjrs}-G_{jm_{1}rs}-G_{km_{1}rs}+G_{jkrs},j=1,\ldots ,m_{1}-1;k\neq j,m_{1}\\ \eta_{1m_{1}}=G_{m_{1}m_{1}rs}-G_{jm_{1}rs}-G_{km_{1}rs}+G_{jkrs},j\neq k\neq m_{1}\\ \eta_{2r}=G_{jkrr}-G_{jkrm_{2}}-G_{jksm_{2}}+G_{jkrs},r=1,\ldots ,m_{2}-1;s\neq r,m_{2}\\ \eta_{2m_{2}}=G_{jkm_{2}m_{2}}-G_{jkm_{2}r}-G_{jkm_{2}s}+G_{jkrs},r\neq s\neq m_{2} \end{array}$

Allele-count	$\begin{array}{c} 2\alpha_{1m_{1}}^{*}+\delta_{1m_{1}}^{*}=0\\ 2\alpha_{2m_{2}}^{*}+\delta_{2m_{2}}^{*}=0 \end{array}$	$\begin{array}{c} {\pi^{\prime }}_{0}=G_{m_{1}m_{1}m_{2}m_{2}}\\ {\pi^{\prime }}_{1j}=\frac{G_{jm_{1}rs}-G_{m_{1}m_{1}rs}-G_{km_{1}rs}+G_{jkrs}}{2},j=1,\ldots ,m_{1}-1;k\neq j,m_{1}\\ {\pi^{\prime }}_{1m_{1}}=-\frac{G_{m_{1}m_{1}rs}-G_{jm_{1}rs}-G_{km_{1}rs}+G_{jkrs}}{2},j\neq k\neq m_{1}\\ {\pi^{\prime }}_{2r}=\frac{G_{jkrm_{2}}-G_{jkm_{2}m_{2}}-G_{jksm_{2}}+G_{jkrs}}{2},r=1,\ldots ,m_{2}-1;r\neq s,m_{2}\\ {\pi^{\prime }}_{2m_{2}}=-\frac{G_{jkm_{2}m_{2}}-G_{jkrm_{2}}-G_{jksm_{2}}+G_{jkrs}}{2},r\neq s\neq m_{2}\\ {\eta^{\prime }}_{1j}=G_{jjrs}-G_{m_{1}m_{1}rs},j=1,\ldots ,m_{1}-1\\ {\eta^{\prime }}_{2r}=G_{jkrr}-G_{jkm_{2}m_{2}},r=1,\ldots ,m_{2}-1 \end{array}$

As pointed out in [9], the genetic effects of a marker may have different interpretation depending upon whether the marker is fitted in a one-locus model or a two-locus model. From the linear model theory, the genetic effects of a marker in a one-locus model are defined based on the expected genotypic values of certain genotypes at this particular marker locus with genotypes at the other marker loci being averaged out based on the joint genotype distribution. For instance, marker 1 in the two-locus setting above has its effects defined in a one-locus model based on the one-locus genotypic values $E\left(G_{jk}\right)=E\left(G_{jkrs}|A_{1j}A_{1k}\right)=\sum_{rs}P\left(A_{2r}A_{2s}|A_{1j}A_{1k}\right)G_{jkrs}$, which could depend on the LDs of alleles between the two loci. When the same marker is fitted in a two-locus model, its effects are usually functions of the expected genotypic values with their joint genotypes taking certain reference alleles or genotypes at the other marker loci. So, in general, even without locus-by-locus interactions, a single marker's effects could be different from the one defined in a multi-locus model when the alleles at different loci are in linkage disequilibrium (LD). Consider a 2-locus haploid model with alleles *A*, *a *at locus 1 and *B*, *b *at locus 2. If we ignore the locus-by-locus interaction, it is easy to show that the additive allelic effects are *α*_1 _= *G_AB _*- *G_aB _*= *G_Ab _*- *G_ab _*and *α*_2 _= *G_AB _*- *G_Ab _*= *G_aB _*- *G_ab _*at locus 1 and 2, respectively. In a one-locus model at locus 1, however, we can show that the locus has its additive allelic effect $\alpha_{1}^{*}=\alpha_{1}+D\alpha_{2}∕\left(p_{A}p_{a}\right)$, where *D *= *P_AB _*- *p_A_p_B _*is the LD between the two loci.

## Simulation Examples

We use some numerical examples to illustrate properties of the models we have discussed. First, we consider the same example discussed in [11] of a three-allele locus with allele frequencies *p*_1 _= 0.2 for *A*_1_, *p*_2 _= 0.3 for *A*_2_, and *p*_3 _= 0.5 for *A*_3_. The six genotypic values are *G*_11 _= 10, *G*_12 _= 30, *G*_22 _= 50, *G*_13 _= 36, *G*_23 _= 46 and *G*_33 _= 42. We adopt a similar strategy to specify the genotype frequencies as: $P_{jj}=p_{j}^{2}-D$ for *j *= 1, 2, 3 and *P_jk _*= 2*p_j_pk *+ *D *for *j *≠ *k*, where D is a measure of departure from Hardy-Weinberg equilibrium (HWE) for the three alleles at the locus and *D*^- ^≤ *D *≤ *D*^+ ^with

$$D^{-}=-\underset{j\neq k}{\min}\left\{2p_{j}p_{k}\right\}=-0.12$$

and

$$D^{+}=\underset{j=1,2,3}{\min}\left\{p_{j}^{2}\right\}=0.04$$

We consider two cases: i) D = 0 for HWE, and ii) *D *= 0.02 for Hardy-Weinberg disequilibrium (HWD). The phenotypic value of an individual is simulated as a sum of its true genotypic value and an environmental noise from *N*(0, *σ*^2^), where the *σ*^2 ^is chosen to be either 0 or *σ*^2 ^= 288 with the latter one corresponds to a 20% heritability level when D = 0. For each of the four configurations, we simulate 10,000 random samples with 1000 individuals each. For each random sample, we fit the three fully parameterized one-locus models (4), (5) and (6) under model framework (2) using the least square approach and estimate the model parameters as well as the six genotypic values. The means and standard deviations (SD) of the least square estimates (LSE) of the model parameters and the six genotypic values from the 10,000 random samples in fitting these three models are summarized in Table 8.

**Table 8:** Means (SD) of LSE for three one-locus models (4), (5) and (6) when *m *= 3.

Allele	*μ*	*α*_1_	*α*_2_	*δ*_11_	*δ*_22_	*δ*_12_	*σ* ^2^
True	42	-6	4	-20	0	-10	
*D *= 0, *σ*^2 ^= 0	42.0(0.00)	-6.00(0.00)	4.00(0.00)	-20.00(0.00)	0.00(0.00)	-10.00(0.00)	0.00(0.00)
*D *= 0, *σ*^2 ^= 288	41.99(1.07)	-5.98(1.61)	3.99(1.44)	-20.06(3.80)	0.02(2.85)	-9.98(2.42)	287.84(12.91)
*D *= 0.02, *σ*^2 ^= 0	42.00(0.00)	-6.00(0.00)	4.00(0.00)	-20.00(0.00)	0.00(0.00)	-10.00(0.00)	0.00(0.00)
*D *= 0.02, *σ*^2 ^= 288	41.98(1.14)	-5.97(1.60)	4.01(1.46)	-20.07(6.21)	0.03(3.09)	-10.04(2.31)	287.81(12.91)
	
	*G*_11_	*G*_22_	*G*_33_	*G*_12_	*G*_13_	*G*_23_	
	
	10	50	42	30	36	46	
	10.00(0.00)	50.00(0.00)	42.00(0.00)	30.00(0.00)	36.00(0.00)	46.00(0.00)	
	9.96(2.73)	49.99(1.79)	41.99(1.07)	30.02(1.55)	36.01(1.20)	45.98(0.98)	
	10.00(0.00)	50.00(0.00)	42.00(0.00)	30.00(0.00)	36.00(0.00)	46.00(0.00)	
	9.96(5.66)	50.03(2.21)	41.98(1.14)	29.97(1.38)	36.01(1.12)	45.99(0.93)	

F_∞_	*τ*	*a*_1_	*a*_2_	*d*_11_	*d*_22_	*d*_12_	*σ*^2^

True	30	-16	4	10	0	-10	
*D *= 0, *σ*^2 ^= 0	30.00(0.00)	-16.00(0.00)	4.00(0.00)	10.00(0.00)	0.00(0.00)	-10.00(0.00)	0.00(0.00)
*D *= 0, *σ*^2 ^= 288	29.98(1.64)	-16.01(1.46)	4.00(1.05)	10.03(1.90)	-0.01(1.42)	-9.98(2.42)	287.84(12.91)
*D *= 0.02, *σ*^2 ^= 0	30.00(0.00)	-16.00(0.00)	4.00(0.00)	10.00(0.00)	0.00(0.00)	-10.00(0.00)	0.00(0.00)
*D *= 0.02, *σ*^2 ^= 288	29.99(3.05)	-16.01(2.88)	4.03(1.25)	10.04(3.10)	-0.01(1.54)	-10.04(2.31)	287.81(12.91)
	
	*G*_11_	*G*_22_	*G*_33_	*G*_12_	*G*_13_	*G*_23_	
	
	10	50	42	30	36	46	
	10.00(0.00)	50.00(0.00)	42.00(0.00)	30.00(0.00)	36.00(0.00)	46.00(0.00)	
	9.96(2.73)	49.99(1.79)	41.99(1.07)	30.02(1.55)	36.01(1.20)	45.98(0.98)	
	10.00(0.00)	50.00(0.00)	42.00(0.00)	30.00(0.00)	36.00(0.00)	46.00(0.00)	
	9.96(5.66)	50.03(2.21)	41.98(1.14)	29.97(1.38)	36.01(1.12)	45.99(0.93)	

Allele-count	*π*_0_	*π*_1_	*π*_2_	*η*_11_	*η*_22_	*η*_12_	*σ*^2^

True	42	-6	4	-32	8	-10	
*D *= 0, *σ*^2 ^= 0	42.00(0.00)	-6.00(0.00)	4.00(0.00)	-32.00(0.00)	8.00(0.00)	-10.00(0.00)	0.00(0.00)
*D *= 0, *σ*^2 ^= 288	41.99(1.07)	-5.98(1.61)	3.99(1.44)	-32.03(2.92)	8.00(2.09)	-9.98(2.42)	287.84(12.91)
*D *= 0.02, *σ*^2 ^= 0	42.00(0.00)	-6.00(0.00)	4.00(0.00)	-32.00(0.00)	8.00(0.00)	-10.00(0.00)	0.00(0.00)
*D *= 0.02, *σ*^2 ^= 288	41.98(1.14)	-5.97(1.60)	4.01(1.46)	-32.02(5.76)	8.05(2.51)	-10.04(2.31)	287.81(12.91)
	
	*G*_11_	*G*_22_	*G*_33_	*G*_12_	*G*_13_	*G*_23_	
	
	10	50	42	30	36	46	
	10.00(0.00)	50.00(0.00)	42.00(0.00)	30.00(0.00)	36.00(0.00)	46.00(0.00)	
	9.96(2.73)	49.99(1.79)	41.99(1.07)	30.02(1.55)	36.01(1.20)	45.98(0.98)	
	10.00(0.00)	50.00(0.00)	42.00(0.00)	30.00(0.00)	36.00(0.00)	46.00(0.00)	
	9.96(5.66)	50.03(2.21)	41.98(1.14)	29.97(1.38)	36.01(1.12)	45.99(0.93)	

As each of the three models provides a re-parameterization of the six genotypic values, for each random sample the three models always give exactly the same estimates of the six genotypic values and the residual variance as we expected, even though their model parameters are defined in different ways. As a result, under each configuration, the three models have the same means and SD for the LSE of the six genotypic values and the residual variance. Without environmental variation, each model can accurately estimate its model parameters and the six genotypic values for each random sample regardless of whether there is HWE or HWD. When there is environmental variation on the phenotypes, it is known that the least square estimators of the model parameters are unbiased under either HWE or HWD. However, the HWD may affect the variance of the least square estimators of the model parameters and the six genotypic values. Note that the genotypic frequencies are *P*_11 _= 0.04, *P*_22 _= 0.09, *P*_33 _= 0.25, *P*_12 _= 0.12, *P*_13 _= 0.20 and *P*_23 _= 0.30 under HWE, while with D = 0.02 the genotypic frequencies become *P*_11 _= 0.02, *P*_22 _= 0.07, *P*_33 _= 0.23, *P*_12 _= 0.14, *P*_13 _= 0.22 and *P*_23 _= 0.32. So, under HWD, we tend to have more individuals carrying genotypes *A*_1_*A*_2_, *A*_1_*A*_3_, *A*_2_*A*_3 _but less individuals carrying genotypes *A*_1_*A*_1_, *A*_2_*A*_2_, *A*_3_*A*_3 _in the random samples than that under HWE. Without knowing the accurate genotypic values, more individuals with certain genotypes in a random sample can then provide better estimates of the corresponding genotypic values. This explains why under HWD the estimates of *G*_11_, *G*_22 _and *G*_33 _have larger SD (or variances) than that under the HWE, and the estimates of *G*_12_, *G*_13 _and *G*_23 _under HWD have smaller variances than that under the HWE.

As another example, let us consider the statistical modeling of two-locus genotypic values *G_jkrs_*, where the first locus have three alleles *A*_1_, *A*_2_, *A*_3 _and the second locus have two alleles *B*_1_, *B*_2_. Assume that the alleles at locus 1 have the same allele frequencies as that in the previous example; i.e., *p*_1 _= 0.2 for *A*_1_, *p*_2 _= 0.3 for *A*_2_, and *p*_3 _= 0.5 for *A*_3_, while the two alleles at locus 2 have frequencies *q*_1 _= 0.2 for *B*_1 _and *q*_2 _= 0.8 for *B*_2_. The two-locus genotypic values *G*_2 _= (*G_jkrs_*), *j*, *k *= 1, 2, 3; *r*, *s *= 1, 2 are given by

$$\begin{array}{c} G_{2}\;=\;\left[\begin{array}{ccc} G_{1111} & G_{1112} & G_{1122}\\ G_{2211} & G_{2212} & G_{2222}\\ G_{3311} & G_{3312} & G_{3322}\\ G_{1211} & G_{1212} & G_{1222}\\ G_{1311} & G_{1312} & G_{1322}\\ G_{2311} & G_{2312} & G_{2322} \end{array}\right]\\ =\;\left[\begin{array}{ccc} 10 & 10.9 & 9.6\\ 50 & 50.3 & 49.9\\ 42 & 42.6 & 41.2\\ 30 & 30.5 & 29.6\\ 36 & 36.8 & 35.4\\ 46 & 46.7 & 45.2 \end{array}\right] \end{array}$$

which are modified values from the previous one-locus model in a way that the *G*_*jk*11 _= *G_jk_*, *G*_*jk*12 _= *G_jk _*+ *e*_1*jk*_and *G*_*jk*22 _= *G_jk _*- *e*_2*jk*_with *e*_1*jk*_and *e*_2*jk*_being some small positive fluctuations according to the genotypes *B*_1_*B*_2 _and *B*_2_*B*_2 _at locus 2. We assume Hardy-Weinberg equilibria at both loci and specify their haplotype frequencies as: *h*_11 _= *p*_1_*q*_1 _- *D*_1_, *h*_12 _= *p*_1_*q*_2 _+ *D*_1_, *h*_21 _= *p*_2_*q*_1 _- *D*_2_, *h*_22 _= *p*_2_*q*_2 _- *D*_2_, *h*_31 _= *p*_3_*q*_1 _+ (*D*_1 _- *D*_2_), *h*_32 _= *p*_3_*q*_2 _- (*D*_1 _- *D*_2_), where *D*_1 _(and *D*_2_) are the linkage disequilibria (LD) between alleles *A*_1 _and *B*_2 _(and *A*_2 _and *B*_1_) at the two loci. We consider two scenarios: i) *D*_1 _= *D*_2 _= 0 for linkage equilibrium (LE); and ii) *D*_1 _= 0, *D*_2 _= 0.03 for LD. The phenotypic value of an individual is still simulated as a sum of its genotypic value and an environmental noise from *N*(0, *σ*^2^), where the *σ*^2 ^was chosen to be either 0 or *σ*^2 ^= 286 with the latter one corresponds to a 20% heritability level when *D*_1 _= *D*_2 _= 0. For each of the four configurations, we simulate 10,000 random samples with 1000 individuals each. For each random sample, we consider fitting models under three model frameworks: i) one-locus models (4), (5) and (6) at locus 1 under model framework (2); ii) two-locus models without epistases from the three coding schemes under model framework (14); iii) fully parameterized two-locus models (13), (14) and (15) with epistases. Still, for each random sample, the three allele coding models under the same model framework give exactly the same estimates of the 18 genotypic values as we expected (results not shown here). As the result, under each model framework, the three models have the same means and SD for the LSE of the 18 genotypic values and the residual variance, although the means and SD for the LSE of their model parameters are different. To compare the LSE of model parameters for models from the same coding under different model frameworks, we summarize in Table 9 the means and SD of the LSE of the model parameters from the 10,000 random samples in fitting the three allele-coding models: the one-locus model (4), the two-locus model under model framework (14),  and the two-locus model under model framework (13). Models from the other two coding schemes behave similarly.

**Table 9:** Means (SD) of LSE for three allele-coding models regarding the two-locus genotypic values

One-locus model	*μ*	*α*_11_	*α*_12_	*δ*_111_	*δ*_122_	*δ*_112_	*σ* ^2^
True	41.68	-5.81	4.03	-20.03	0.29	-10	
*D*_1 _= *D*_2 _= 0, *σ*^2 ^= 0	41.68(0.04)	-5.81(0.06)	4.03(0.06)	-20.03(0.14)	0.29(0.09)	-10.00(0.08)	0.37(0.01)
*D*_1 _= *D*_2 _= 0, *σ*^2 ^= 286	41.69(1.07)	-5.83(1.61)	4.03(1.44)	-20.00(3.79)	0.27(2.85)	-9.99(2.43)	286.58(12.82)

True	41.55	-5.74	4.21	-20.04	0.09	-10.06	
*D*_1 _= 0, *D*_2 _= 0.03, *σ*^2 ^= 0	41.55(0.04)	-5.74(0.06)	4.21(0.06)	-20.04(0.14)	0.09(0.09)	-10.06(0.08)	0.36(0.01)
*D*_1 _= 0, *D*_2 _= 0.03, *σ*^2 ^= 286	41.54(1.07)	-5.74(1.61)	4.23(1.45)	-20.09(3.81)	0.07(2.83)	-10.09(2.43)	286.27(12.94)

Two-locus model - no epistases	*μ*	*α*_11_	*α*_12_	*δ*_111_	*δ*_122_	*δ*_112_	*α*_21_

True	41.88	-5.81	4.03	-20.03	0.29	-10	0.64
*D*_1 _= *D*_2 _= 0, *σ*^2 ^= 0	41.88(0.02)	-5.81(0.01)	4.03(0.01)	-20.03(0.01)	0.29(0.05)	-10.00(0.02)	0.64(0.02)
*D*_1 _= *D*_2 _= 0, *σ*^2 ^= 286	41.91(2.88)	-5.82(1.61)	4.03(1.44)	-19.99(3.79)	0.27(2.85)	-9.99(2.43)	0.63(2.90)
	
	*δ*_211_	*σ*^2^					
	
	-1.92						
	-1.92(0.03)	0.024(0.002)					
	-1.92(3.41)	285.64(12.79)					

True	41.85	-5.80	4.06	-20.04	0.14	-10.04	0.65
*D*_1 _= 0, *D*_2 _= 0.03, *σ*^2 ^= 0	41.85(0.02)	-5.80(0.01)	4.06(0.01)	-20.04(0.01)	0.14(0.05)	-10.04(0.02)	0.65(0.02)
*D*_1 _= 0, *D*_2 _= 0.03, *σ*^2 ^= 286	41.87(2.94)	-5.80(1.61)	4.07(1.45)	-20.09(3.81)	0.12(2.83)	-10.07(2.43)	0.62(2.88)
	
	*δ*_211_	*σ*^2^					
	
	-1.92						
	-1.92(0.03)	0.02(0.00)					
	-1.88(3.38)	285.36(12.94)					

Two-locus model with epistases	*μ*	*α*_11_	*α*_12_	*δ*_111_	*δ*_122_	*δ*_112_	*α*_21_

True	42	-6	4	-20	0	-10	0.6
*D*_1 _= *D*_2 _= 0, *σ*^2 ^= 0	42.00(0.00)	-6.00(0.00)	4.00(0.00)	-20.00(0.00)	0.00(0.00)	-10.00(0.00)	0.60(0.00)
*D*_1 _= *D*_2 _= 0, *σ*^2 ^= 286	41.92(5.73)	-5.99(8.65)	4.04(7.67)	-19.79(19.86)	-0.04(15.62)	-9.82(13.54)	0.66(6.04)
*D*_1 _= 0, *D*_2 _= 0.03, *σ*^2 ^= 0	42.00(0.00)	-6.00(0.00)	4.00(0.00)	-20.00(0.00)	0.00(0.00)	-10.00(0.00)	0.60(0.00)
*D*_1 _= 0, *D*_2 _= 0.03, *σ*^2 ^= 286	42.24(8.60)	-6.11(11.83)	3.66(10.05)	-20.04(22.95)	0.51(14.85)	-9.77(14.64)	0.38(8.86)
	
	*δ*_211_	(*α*_11_*α*_21_)	(*α*_12_*α*_21_)	(*δ*_111_*α*_21_)	(*δ*_122_*α*_21_)	(*δ*_112_*α*_21_)	(*α*_11_*δ*_211_)
	
	-2	0.2	0.1	-0.1	-0.5	-0.4	-0.2
	-2.00(0.00)	0.20(0.00)	0.10(0.00)	-0.10(0.00)	-0.50(0.00)	-0.40(0.00)	-0.20(0.00)
	-2.05(6.99)	0.23(9.12)	0.07(8.08)	-0.35(20.98)	-0.47(16.35)	-0.65(14.30)	-0.29(10.58)
	-2.00(0.00)	0.20(0.00)	0.10(0.00)	-0.10(0.00)	-0.50(0.00)	-0.40(0.00)	-0.20(0.00)
	-1.80(9.71)	0.24(12.24)	0.39(10.44)	-0.03(24.03)	-0.94(15.63)	-0.52(15.27)	-0.15(13.52)
	
	(*α*_12_*δ*_211_)	(*δ*_111_*δ*_211_)	(*δ*_122_*δ*_211_)	(*δ*_112_*δ*_211_)	*σ*^2^		
	
	-0.2	0.2	1.7	1			
	-0.20(0.00)	0.20(0.00)	1.70(0.00)	1.00(0.00)	0.00(0.00)		
	-0.18(9.39)	0.55(24.51)	1.70(18.94)	1.35(16.46)	282.45(12.83)		
	-0.20(0.00)	0.20(0.00)	1.70(0.00)	1.00(0.00)	0.00(0.00)		
	-0.44(11.64)	0.07(27.44)	2.11(18.14)	0.98(17.29)	282.81(12.74)		

As we mentioned before, the one-locus models are actually modeling the expected genotypic values given the genotypes at locus 1. When *D*_1 _= *D*_2 _= 0, we can show that the expected genotypic values at locus 1 are *G*_11 _= 10.03, *G*_22 _= 50.03, *G*_33 _= 41.68, *G*_12 _= 29.90, *G*_13 _= 35.87 and *G*_23 _= 45.71, which correspond to *μ *= 41.68, *α*_11 _= -5.81, *α*_12 _= 4.03, *δ*_111 _= -20.03, *δ*_122 _= 0.29 and *δ*_112 _= -10 as the true parameters in the allele coding one-locus model. When *D*_1 _= 0, *D*_2 _= 0.03, the expected genotypic values at locus 1 become *G*_11 _= 10.03, *G*_22 _= 50.08, *G*_33 _= 41.55, *G*_12 _= 29.97, *G*_13 _= 35.81 and *G*_23 _= 45.77, which correspond to *μ *= 41.55, *α*_11 _= -5.74, *α*_12 _= 4.21, *δ*_111 _= -20.04, *δ*_122 _= 0.09 and *δ*_112 _= -10.06 as the true parameters in the allele coding one-locus model. In both cases, the least square estimators of the one-locus model parameters are unbiased estimators of the true parameters. Note that, unlike the one-locus model in the previous example, the LSE of the model parameters are no longer exactly the same as the true values even when no environmental noises are involved. The reason is that the expected genotypic values at locus 1 depend on not only the genotypic values but also the joint genotype frequencies in the sample, which may change slightly from sample to sample due to the sampling variation.

For the two-locus model without epistases, it cannot provide unbiased estimators for all the genotypic values because of the model mis-specification. However, the LSE of its parameters associated with locus 1 are similar to the ones in the one-locus model at locus 1. In fact, as we know from the linear model theory, the true values of its parameters associated with locus 1 are the same as the ones defined in the one-locus model at locus 1 when the two loci are in LE. Under LD, the least square estimators of its model parameters associated with locus 1 could be biased, and the biasness depends on the LD setting.

The two-locus model with epistases gives a full re-parameterization of the 18 genotypic values. Therefore, when no environmental noises are involved, the LSE of its model parameters are exactly the same as their true values for each random sample regardless of the LD between the two loci. It has to be pointed out that this phenomenon holds only when the random sample contains all the 18 possible genotypes. In our simulation setting, the frequencies for certain genotypes such as *A*_1_*A*_1_*B*_1_*B*_1_, *A*_1_*A*_3_*B*_1_*B*_1 _and *A*_2_*A*_2_*B*_1_*B*_1 _are pretty small. As the result, we occasionally (about 22-23% of the 1000 random samples) may obtain a random sample that has no individuals carrying certain genotypes. In this case, the design matrix in the fully parameterized model becomes singular and the LSE of the model parameters are no longer unique. To keep our illustration of the model properties simple, we excluded those random samples in fitting the two-locus model with epistases (reduced models are less likely to have singular design matrices). Other techniques such as ridge regression could be applied to handle those skewed random samples. In the presence of environmental noises, it is also noted that the LSE for some of its model parameters such as *δ*_111_, (*δ*_111_*α*_21_) and (*δ*_111_*δ*_211_) have much larger SD than the LSE of other parameters. This is due to the low frequencies of genotypes *A*_1_*A*_1_*B*_1_*B*_1_, *A*_1_*A*_3_*B*_1_*B*_1 _and *A*_2_*A*_2_*B*_1_*B*_1_. As a random sample has few individuals carrying these genotypes, it has reduced accuracy in estimation of their corresponding true genotypic values to which the model parameters *δ*_111_, (*δ*_111_*α*_21_) and (*δ*_111_*δ*_211_) are related.

## Discussion

In this study, we introduced three genotype coding schemes to build F_∞ _models for multi-allele markers. The relationship between the model parameters and the expected genotypic values were established in some fully parameterized as well as reduced one-locus and two-locus F_∞ _models. Our results showed that the relationships between the model parameters and the expected genotypic values could become more intricate in the multi-allele case than that in the biallelic case, even though the extension of the coding schemes from biallelic to multiple alleles appears straightforward. We built the relationships between different model parameters mainly through their coding variables of marker genotypes, which simplified the tedious derivation process comparing with the classical matrix approach. The F_∞ _models we proposed can be used directly for association testing of multi-allele markers and their possible interactions with quantitative traits using random unrelated samples. These F_∞ _models could also be applied to test for the risk haplotypes and their interactions when incorporated with the likelihood approach (e.g., [20]), or analyze family data by combining them with the likelihood to account for the transmission probability of alleles from parents to their offspring. Although our discussion focused on genetic modeling of quantitative traits, the results can be extended to other phenotypic traits such as binary outcomes in case-control studies using logistic regression models or time-to-event data using the Cox proportional hazard models.

Throughout the paper, we assumed that all the possible genotypes are available from the sampled individuals. If certain genotypes are not observable, then the expected genotypic values on these genotypes will not be estimable by themselves, which could change the interpretation of the model parameters as well. The models we have presented can also be modified to handle the situation when some individuals have missing genotypes at certain marker loci. When the missing genotypes at a marker locus have both alleles missing at the same time, we can simply introduce an indicator variable to code for the missing genotype at the marker. The regression coefficient of this indicator variable for this missing genotype can usually be interpreted as the difference between the expected genotypic value with missing genotype at the marker locus and the intercept of the model, while the other regression coefficients would keep the same interpretation as before.

It has to be pointed out that the relationships between the model parameters and the expected genotypic values are based on the assumption that the models can correctly specify the structure of the expected genotypic values. When a fully parameterized model is applied, the definition of its model parameters do not depend on the allele frequencies, HWD among alleles within a locus, or LD structure between alleles at different loci. In fitting a reduced model, however, a simplified model may not be totally correct in modeling all the expected genotypic values. In this case, depending on how accurate the simplified model is on approximating the expected genotypic values, the allele frequencies, HWD and LD structure between marker alleles could affect the definition and LSE of its model parameters. In the presence of environmental variation on the phenotypic values, regardless of whether a fully parameterized or reduced model is applied, the allele frequencies, HWD or LD between marker alleles may affect the LSE of the model parameters and the power in detection of the associated marker alleles as shown in our simulation studies.

All the models we have discussed so far are F_∞ _models. Statistically, these F_∞ _models are fixed-effect models which focus on modeling the expected genotypic values directly. On the other hand, the Fisher's ANOVA models, which target on evaluation of the variations contributed by various allelic effects and interactions, can be treated as random-effect models (see [21]) in which the expected genotypic values come from a discrete random variable *G*(*g*) = E(*G*|*g*) with its limited genotypes *g *being randomly sampled from a study population. Both the F_∞ _and the Fisher type models form basis in the analysis of quantitative traits and they provide different perspectives in assessing the genetic effects of QTL and markers. For biallelic markers, we proposed in [10] a 'mean corrected' Fisher (mc-Fisher) model for decomposition of the genotypic variances. In the multi-allele marker case, we can also construct similar mc-Fisher models by applying mean corrections on all the indicator variables of the paternal and maternal alleles in the allele coding F_∞ _models. For example, based on the allele coding model (4), we can construct its corresponding mc-Fisher model by replacing the coding variables *w_j _*and *v_jk _*with ${\bar{w}}_{j}=w_{j}-2p_{j}$ and ${\bar{v}}_{jk}=\left(z_{1j}-p_{j}\right)\left(z_{2k}-p_{k}\right)=v_{jk}-\left(p_{j}w_{k}+p_{k}w_{j}\right)/2+p_{j}p_{k}$, respectively; where *p_j _*is the allele frequency of *A_j_*. Then the genetic additive and dominant variance components *V_A _*and *V_D _*of *G*(*g*), which are defined as variations contributed by the additive allelic effect and allelic interactions respectively, can be estimated from ${\bar{w}}_{j}$'s and ${\bar{v}}_{jk}$'s separately. As pointed out in [10], the mc-Fisher model can provide an orthogonal partition of V(G) into the sum of *V_A _*and *V_D _*under Hardy-Weinberg equilibrium, and it can be fitted through the standard least-square regression approach. Similar to the F_∞ _models, the definition of the model parameters in such a mc-Fisher model also depend on the choice of the reference allele '*A_m_*'. But the estimates of the additive and dominant variance components *V_A _*and *V_D _*do not depend on such a choice. In addition, when a fully parameterized model is applied, the mc-Fisher model is equivalent to its original F_∞ _model in modeling the expected genotypic values. Therefore, both models have the same residual variance and the F-statistics in testing for the overall effect of the marker locus. When reduced models are applied, the mc-Fisher model could become inequivalent to its original F_∞ _model especially when allelic interactions are involved.

Of the three coding schemes that we have discussed, the F_∞ _coding is perhaps the most widely used in current genetic association studies of quantitative traits. From what we have shown, the three coding schemes can essentially lead to equivalent models and have the same power in detection of various genetic effects. In practice, just like the various existing coding schemes such as 'Reference', 'GLM' and 'Effect' that are commonly used in the analysis of categorical covariates [22], we usually only need to adopt one specific coding scheme in building the regression models. Which coding scheme should be applied depends on how convenient it can provide the statistical inferences on the parameters of our research interests. In general, the allele coding models can provide direct estimates of certain substitution effects of alleles and allelic interactions and, in the two-locus case, allele coding models are perhaps the easiest among the three codings in building the relationships between their model parameters and the expected genotypic values. Besides, they are generically linked to the genetic variance components as we have shown above. On the other hand, the allele-count coding models are attractive in that it often leads to simple comparisons among the three genotypic groups with 0, 1 or 2 copies of a particular allele. In the two-locus case, the allele-count coding models also have the definition of their model parameters remain as simple as (if not simpler than) that in the allele coding models even in the presence of epistases. Meanwhile, both the allele and allele-count coding show an advantage that their lower-order main effects in the models can keep the same interpretation regardless of whether there are epistases involved in the model or not. In contrast, the F_∞ _coding models may have the definition of their lower-order main effects vary depending on the absence or presence of epistases in the models. Even though the one-locus F_∞ _coding model parameters are closely related to the additive and dominance effects, the two-locus F_∞ _coding model parameters including the lower-order main effects have more complicated interpretations than that in the allele or allele-count coding models especially when epistases are involved.

The coding of marker genotypes are not limited to the three allele-based coding schemes that we have discussed. Application of a coding scheme could also be subject to the number of individuals available in each genotype group. For example, under the model framework (7), the allele coding scheme typically creates *w_j_*(*g*), *v_j_*(*g*) for each allele type *A_j_*, *j *= 1, ..., *m*. When the group of a homozygous genotype *A_j_A_j _*includes very few individuals for a particular allele *A_j_*, we may want to combine this genotypic group with another genotype such as the one carrying one copy of the allele *A_j_*. Then we can replace the original *w_j_*(*g*) and *v_j_*(*g*) by an allele presence-absence coding variable *d_j_*(*g*) for this specific allele *A_j _*while keeping two coding variables *w_k_*(*g*), *v_k_*(*g*) for other alleles *A_k_*, which leads to a mixed use of the allele coding and this allele presence-absence coding variable. In certain situations, the genotype-based coding could also be very useful as it can provide direct tests on pair-wise comparisons of certain genotypic values. Comparing with the genotype-based coding, the allele coding has the advantage of further dissecting the genetic effects into the allelic effects and allelic interactions, which allow us to specify reduced models with varying degrees of interactions among the main allelic effects - a useful tool in the model building procedures. Given a fixed coding, the likelihood ratio test can be applied to compare a full model with its reduced models. Statistical model selection tools such as AIC and BIC criteria, which provide a balance between the goodness of model fitting to the data and the complexity of the models in terms of the number of parameters, could also be used to compare some non-nested reduced models or frameworks. The current study focuses on establishing the theoretical relationships between the model parameters and the expected genotypic values according to different coding schemes under various model frameworks. A power comparison of some reduced models from different coding schemes under various scenarios with respect to the allele frequencies and possible HWD or LDs between marker alleles is beyond the scope of this study and might be worth of further exploration.

## Conclusions

In summary, we introduced three allele-based coding schemes to construct F_∞ _models for association testing of multi-allele genetic markers with quantitative traits. Depending upon whether certain allelic effects or comparisons between genotypic groups are of the main research interest, investigators may adopt one of the three allele-based codings (i.e., allele, F_∞ _or allele-count), or perhaps a genotype-based coding in building an F_∞ _model. Based on the F_∞ _model from a given coding scheme, standard regression model fitting tools can then be applied to estimate or test for various genetic effects. Understanding the definition of model parameters from different coding schemes under various model frameworks are crucial for constructing appropriate testing hypothesis and making the correct statistical inferences in the genetic association studies.

## Authors' contributions

TW planned the study, conducted the derivation and wrote the manuscript.

## Appendices

### A. Estimability of parameters in model (8)

Let *G *= (*G*(*g*_1_), ...,*G*(*g_N_*))*^T ^*denote a vector of the expected genotypic values of all the individuals in the sample, and $\beta^{*}=\left(\mu^{*},\alpha_{1}^{*},\;.\;.\;.\;,\alpha_{m}^{*},\delta_{1}^{*},\;.\;.\;.\;,\delta_{m}^{*}\right)$ be a vector of all the model parameters. We can rewrite model (8) in a matrix form as *G *= Xβ* +e, where *e *= (*e*_1_, ..., *e_N_*) and the design matrix X is

(18) $$X=\left[1_{N}W_{1}W_{2}\cdot \cdot \cdot W_{m}V_{1}V_{2}\cdot \cdot \cdot V_{m}\right]$$

with *W_j _*= (*w_j_*(*g*_1_), ..., *w_j_*(*g_N_*))*^T ^*and *V_j _*= (*v_j_*(*g*_1_), ..., *v_j_*(*g_N_*))*^T ^*for *j *= 1, ..., *m*. As every individual carries two and only two alleles at the locus, we have $\sum_{j=1}^{m}W_{j}=2\cdot 1_{N}$, which means that the first (m+1) column vectors 1*_N_*, *W*_1_, *W*_2_, ..., *W_m _*of the design matrix *X *are linearly dependent. So, *rank*(*X*) ≤ 2*m*; i.e., *X *is not a full column rank matrix.

From (7), we have $G_{jk}=\mu^{*}+\alpha_{j}^{*}+\alpha_{k}^{*}$ for *j *≠ *k*, and $G_{jj}=\mu^{*}+2\alpha_{j}^{*}+\delta_{j}^{*}$. If we write *G*_0 _= (*G*_12_, *G*_13_, ..., *G*_1*m*_, *G*_23_, ..., *G*_*L*-1, *L*_, *G*_11_, ..., *G_mm_*)*^T^*, then this model gives

$$G_{0}=X_{0}\beta^{*}=\left[\begin{array}{ccc} 1_{s} & X_{A} & 0_{s\times m}\\ 1_{m} & 2\cdot I_{m\times m} & I_{m\times m} \end{array}\right]\beta^{*}$$

where *s *= *m*(*m *- 1)/2, and

$$X_{A}=\left[\begin{array}{ccccc} 1 & 1 & 0 & \ldots & 0\\ 1 & 0 & 1 & \ldots & 0\\ \vdots & \vdots & \vdots & \ddots & \vdots \\ 1 & 0 & 0 & \ldots & 1\\ 0 & 1 & 1 & \ldots & 0\\ \vdots & \vdots & \vdots & & \vdots \\ 0 & 0 & 0 & \ldots & 1 \end{array}\right]$$

Assume that the genotypes of the sampled individuals cover all possible genotypes *A_j_A_k _*for *j*, *k *= 1, ..., *m*. Then the design matrix *X *includes all the row vectors of *X*_0_, which implies that *rank*(*X*) ≥ *rank*(*X*_0_). It is clear that *rank*(*X*_0_) = *m *+ *rank*([**1***_s _X_A_*]), and it can be shown that *rank*([**1***_s _X_A_*]) = *rank*(*X_A_*) = *m *when *m *≥ 3. Therefore, *rank*(*X*) = 2*m *as *m *≥ 3. Note that when *m *= 2, we have *s *= 1 and *rank*(*X*) = 3.

From the linear models theory, we know that for a vector *λ *= (*λ*_0_, ..., *λ*_2*m*_)*^T ^*∈ *R*^2*m*+1^, a linear function λ^T^β* of β* is estimable if and only if λ⊥N(X), where $N\left(X\right)=\left\{c\in R^{2m+1}|Xc=0\right\}$ is the null space of the design matrix *X*. It is also known that $N\left(X\right)⊕ℛ\left(X\right)=R^{2m+1}$, where ℛ(X) is a linear space generated by the row vectors of *X*. Hence, we have rank(N(X))=(2m+1)-rank(ℛ(X))=1. Note that $c={\left(2,-1_{m}^{\prime },0_{m}^{\prime }\right)}^{\prime }\in N\left(X\right)$ due to the linear dependency among the column vectors 1*_N_*, *W*_1_, *W*_2_, ..., *W_m _*in the design matrix *X*. Therefore, for a vector *λ *= (*λ*_0_, *λ*_1_, ..., *λ*_2*m*_)*^T ^*∈ *R*^2*m*+1^, the linear function λ^T^β* is estimable if and only if *λ *⊥ *c*, or equivalently, $2\lambda_{0}=\sum_{j=1}^{m}\lambda_{j}$. As a result, we know that in model (8) the functions of model parameters $G_{jk}=\mu^{*}+\alpha_{j}^{*}+\alpha_{k}^{*}$ for *j *≠ *k*, and $G_{jj}=\mu^{*}+2\alpha_{j}^{*}+\delta_{j}^{*}$ for *j *= 1, ..., *m *are estimable, and the parameters $\delta_{j}^{*}=G_{jj}-\left(\mu^{*}+2\alpha_{j}^{*}\right)=G_{jj}+G_{kl}-G_{jl}-G_{jk}$ as *j *≠ *k*, *l *and *k *≠ *l *(or in abbreviation, *j *≠ *k*, ≠ *l*) for *j *= 1, ..., *m *are also estimable. But the parameters μ* and $\alpha_{1}^{*},\;.\;.\;.\;,\alpha_{m}^{*}$ themselves are not estimable.

### B. Estimability of parameters in model (9)

For model (9), we have its design matrix

$$W=\left[1_{N}W_{1}W_{2}\cdot \cdot \cdot W_{m-1}V_{1}V_{2}\cdot \cdot \cdot V_{m}\right]$$

where *W_j _*= (*w_j_*(*g*_1_), ..., *w_j_*(*g_N_*))*^T ^*and *V_j _*= (*v_j_*(*g*_1_), ..., *v_j_*(*g_N_*))*^T ^*for *j *= 1, ..., *m*. It can be shown that the *W *and the design matrix *X *defined in (16) for model (8) have the following relationship *W *= *XT *or *X *= *WS^T^*, where

$$T=\left[\begin{array}{cc} I_{m} & 0\\ 0_{1\times m} & 0_{1\times m}\\ 0 & I_{m} \end{array}\right]$$

and

$$S^{T}=\left[\begin{array}{ccc} I_{m} & d & 0_{m\times m}\\ 0_{1\times m} & 0 & 0_{1\times m}\\ 0_{m\times m} & 0_{m\times 1} & I_{m} \end{array}\right]$$

with *d *= (2, -1, ..., -1)' ∈ *R^m^*. Let *β *= (*μ*, *α*_1_, ..., *α*_*m*-1_, *δ*_1_, ..., *δ_m_*). Therefore, as (8) and (9) are two equivalent models, we have *G *= Xβ* = WS^T^β* = *Wβ*, which yields

$$\beta =\left[\begin{array}{c} \mu \\ \alpha_{1}\\ \vdots \\ \alpha_{m-1}\\ \delta_{1}\\ \vdots \\ \delta_{m} \end{array}\right]=S^{T}\beta^{*}=\left[\begin{array}{c} \mu^{*}+2\alpha_{m}^{*}\\ \alpha_{1}^{*}-\alpha_{m}^{*}\\ \vdots \\ \alpha_{m-1}^{*}-\alpha_{m}^{*}\\ \delta_{1}^{*}\\ \vdots \\ \delta_{m}^{*} \end{array}\right]$$

From this relationship, we have $\delta_{j}=\delta_{j}^{*}$, *j *= 1, ..., *m*, which are estimable as shown in Appendix A. Besides, the intercept $\mu =\mu^{*}+2\alpha_{m}^{*}=G_{jm}+G_{km}-G_{jk}$ and $\alpha_{j}=\alpha_{j}^{*}-\alpha_{m}^{*}=G_{jk}-G_{km}$, *k *≠ *j*, *j *= 1, ..., *m *- 1, are also estimable.

### C. Relationships for fully parameterized two-locus models

**(C.1) **Relationships between parameters of the fully parameterized two-locus model (13) and the expected genotypic values are

$$\{\begin{array}{l} \mu =G_{m_{1}m_{1}m_{2}m_{2}}\\ \alpha_{1j}=G_{jm_{1}m_{2}m_{2}}-\mu =G_{jm_{1}m_{2}m_{2}}-G_{m_{1}m_{1}m_{2}m_{2}}\\ \alpha_{2r}=G_{m_{1}m_{1}rm_{2}}-\mu =G_{m_{1}m_{1}rm_{2}}-G_{m_{1}m_{1}m_{2}m_{2}}\\ \delta_{1jk}=G_{jkm_{2}m_{2}}-\alpha_{1j}-\alpha_{1k}-\mu \\ =G_{jkm_{2}m_{2}}-(G_{jm_{1}m_{2}m_{2}}+G_{m_{1}km_{2}m_{2}})\\ +G_{m_{1}m_{1}m_{2}m_{2}}\\ \delta_{2rs}=G_{m_{1}m_{1}rs}-\alpha_{2r}-\alpha_{2s}-\mu \\ =G_{m_{1}m_{1}rs}-(G_{m_{1}m_{1}rm_{2}}+G_{m_{1}m_{1}sm_{2}})\\ +G_{m_{1}m_{1}m_{2}m_{2}}\\ (\alpha_{1j}\alpha_{2r})=G_{jm_{1}rm_{2}}-\alpha_{1j}-\alpha_{2r}-\mu \\ =G_{jm_{1}rm_{2}}-(G_{jm_{1}m_{2}m_{2}}+G_{m_{1}m_{1}rm_{2}})\\ +G_{m_{1}m_{1}m_{2}m_{2}}\\ (\delta_{1jk}\alpha_{2r})=G_{jkrm_{2}}-\alpha_{1j}-\alpha_{1k}-\delta_{1jk}\\ -\alpha_{2r}-(\alpha_{1j}\alpha_{2r})-(\alpha_{1k}\alpha_{2r})-\mu \\ G_{jkrm_{2}}-(G_{jkm_{2}m_{2}}+G_{jm_{1}rm_{2}}\\ +G_{km_{1}rm_{2}})+(G_{jm_{1}m_{2}m_{2}}+G_{km_{1}m_{2}m_{2}}\\ +G_{m_{1}m_{1}rm_{2}})-G_{m_{1}m_{1}m_{2}m_{2}}\\ (\alpha_{1j}\delta_{2rs})=G_{jm_{1}rs}-\alpha_{2r}-\alpha_{2s}-\delta_{2rs}\\ -\alpha_{1j}-(\alpha_{1j}\alpha_{2r})-(\alpha_{1j}\alpha_{2s})-\mu \\ G_{jm_{1}rs}-(G_{m_{1}m_{1}rs}+G_{jm_{1}rm_{2}}\\ +G_{jm_{1}sm_{2}})+(G_{jm_{1}m_{2}m_{2}}+G_{m_{1}m_{1}rm_{2}}\\ +G_{m_{1}m_{1}sm_{2}})-G_{m_{1}m_{1}m_{1}m_{2}}\\ (\delta_{1jk}\delta_{2rs})=G_{jkrs}-\alpha_{1j}-\alpha_{1k}-\delta_{1jk}-\alpha_{2r}-\alpha_{2s}\\ -\delta_{2rs}-(\alpha_{1j}\alpha_{2r})-(\alpha_{1j}\alpha_{2s})-(\alpha_{1k}\alpha_{2r})\\ -(\alpha_{1k}\alpha_{2s})-(\alpha_{1j}\delta_{2rs})-(\alpha_{1k}\delta_{2rs})\\ -(\delta_{1jk}\alpha_{2r})-(\delta_{1jk}\alpha_{2s})-\mu \\ =G_{jkrs}-(G_{jm_{1}rs}+G_{km_{1}rs}+G_{jkrm_{2}}\\ +G_{jksm_{2}})+(G_{jkm_{2}m_{2}}+G_{jm_{1}rm_{2}}\\ +G_{km_{1}rm_{2}}+G_{jm_{1}sm_{2}}+G_{km_{1}sm_{2}}\\ +G_{m_{1}m_{1}rs})-(G_{jm_{1}m_{2}m_{2}}+G_{km_{1}m_{2}m_{2}}\\ +G_{m_{1}m_{1}rm_{2}}+G_{m_{1}m_{1}sm_{2}})+G_{m_{1}m_{1}m_{2}m_{2}} \end{array}$$

for *j*, *k *= 1, ..., *m*_1 _- 1; *r*, *s *= 1, ..., *m*_2 _- 1 and *j *≥ *k*, *r *≤ *s*.

**(C.2) **Relationships between parameters of the fully parameterized two-locus model (14) and the expected genotypic values are

$$\{\begin{array}{l} \tau =\mu +\sum_{j=1}^{m_{1}-1}\left(\alpha_{1j}+\frac{\delta_{1jj}}{2}\right)+\sum_{r=1}^{m_{2}-1}\left(\alpha_{2r}+\frac{\delta_{2rr}}{2}\right)\\ +\sum_{j=1}^{m_{1}-1}\sum_{r=1}^{m_{2}-1}\left[(\alpha_{1j}\alpha_{2r}\right)+\frac{\left(\alpha_{1j}\delta_{2rr}\right)}{2}\\ +\frac{\left(\delta_{1jj}\alpha_{2r}\right)}{2}+\frac{\left(\delta_{1jj}\delta_{2rr}\right)}{4}]\\ =\frac{1}{4}\sum_{j=1}^{m_{1}-1}\sum_{r=1}^{m_{2}-1}G_{jjrr}+\frac{\left(3-m_{2}\right)}{4}\sum_{j=1}^{m_{1}-1}G_{jjm_{2}m_{2}}\\ +\frac{\left(3-m_{1}\right)}{4}\sum_{r=1}^{m_{2}-1}G_{m_{1}m_{1}rr}+\frac{\left(3-m_{1})(3-m_{2}\right)}{4}G_{m_{1}m_{1}m_{2}m_{2}}\\ a_{1j}=\alpha_{1j}+\frac{\delta_{1jj}}{2}+\sum_{r=1}^{m_{2}-1}\left[(\alpha_{1j}\alpha_{2r}\right)+\frac{\left(\alpha_{1j}\delta_{2rr}\right)}{2}\\ +\frac{\left(\delta_{1jj}\alpha_{2r}\right)}{2}+\frac{\left(\delta_{1jj}\delta_{2rr}\right)}{4}]\\ =\frac{1}{4}\sum_{r=1}^{m_{2}-1}\left(G_{jjrr}-G_{m_{1}m_{1}rr}\right)\\ +\frac{\left(3-m_{2}\right)}{4}(G_{jjm_{2}m_{2}}-G_{m_{1}m_{1}m_{2}m_{2}})\\ a_{2r}=\alpha_{2r}+\frac{\delta_{2rr}}{2}+\sum_{j=1}^{m_{1}-1}\left[(\alpha_{1j}\alpha_{2r}\right)+\frac{\left(\alpha_{1j}\delta_{2rr}\right)}{2}\\ +\frac{\left(\delta_{1jj}\alpha_{2r}\right)}{2}+\frac{\left(\delta_{1jj}\delta_{2rr}\right)}{4}]\\ =\frac{1}{4}\sum_{j=1}^{m_{1}-1}\left(G_{jjrr}-G_{jjm_{2}m_{2}}\right)\\ +\frac{\left(3-m_{1}\right)}{4}(G_{m_{1}m_{1}rr}-G_{m_{1}m_{1}m_{2}m_{2}})\\ d_{1jj}=-\frac{\delta_{1jj}}{2}-\sum_{r=1}^{m_{2}-1}\left[\frac{\left(\delta_{1jj}\alpha_{2r}\right)}{2}+\frac{\left(\delta_{1jj}\delta_{2rr}\right)}{4}\right]\\ =-\frac{1}{4}\sum_{r=1}^{m_{2}-1}\left(G_{jjrr}-2G_{jm_{1}rr}+G_{m_{1}m_{1}rr}\right)\\ -\frac{\left(3-m_{2}\right)}{4}(G_{jjm_{2}m_{2}}-2G_{jm_{1}m_{2}m_{2}}+G_{m_{1}m_{1}m_{2}m_{2}})\\ d_{2rr}=-\frac{\delta_{2rr}}{2}-\sum_{j=1}^{m_{1}-1}\left[\frac{\left(\alpha_{1j}\delta_{2rr}\right)}{2}+\frac{\left(\delta_{1jj}\delta_{2rr}\right)}{4}\right]\\ =-\frac{1}{4}\sum_{j=1}^{m_{1}-1}\left(G_{jjrr}-2G_{jjrm_{1}}+G_{jjm_{2}m_{2}}\right)\\ -\frac{\left(3-m_{1}\right)}{4}(G_{m_{1}m_{1}rr}-2G_{m_{1}m_{1}rm_{2}}+G_{m_{1}m_{1}m_{2}m_{2}})\\ d_{1jk}=\delta_{1jk}+\sum_{r=1}^{m_{2}-1}\left[(\delta_{1jk}\alpha_{2r})+\frac{\left(\delta_{1jk}\delta_{2rr}\right)}{2}\right]\\ =\frac{1}{2}\sum_{r=1}^{m_{2}-1}\left(G_{jkrr}-G_{jm_{1}rr}-G_{km_{1}rr}+G_{m_{1}m_{1}rr}\right)\\ +\frac{\left(3-m_{2}\right)}{4}(G_{jkm_{2}m_{2}}-G_{jm_{1}m_{2}m_{2}}-G_{km_{1}m_{2}m_{2}}\\ +G_{m_{1}m_{1}m_{2}m_{2}}),j<k\\ d_{2rs}=\delta_{2rs}+\sum_{j=1}^{m_{1}-1}\left[(\alpha_{1j}\delta_{2rs})+\frac{\left(\delta_{1jj}\delta_{2rr}\right)}{2}\right]\\ =\frac{1}{2}\sum_{j=1}^{m_{1}-1}\left(G_{jjrs}-G_{jjrm_{2}}-G_{jjsm_{2}}+G_{jjm_{2}m_{2}}\right)\\ +\frac{\left(3-m_{1}\right)}{4}(G_{m_{1}m_{1}rr}-G_{m_{1}m_{1}rm_{2}}-G_{m_{1}m_{1}sm_{2}}\\ +G_{m_{1}m_{1}m_{2}m_{2}}),r<s\\ (a_{1j}a_{2r})=(\alpha_{1j}\alpha_{2r})+\frac{\left(\alpha_{1j}\delta_{2rr}\right)}{2}+\frac{\left(\delta_{1jj}\alpha_{2r}\right)}{2}+\frac{\left(\delta_{1jj}\delta_{2rr}\right)}{4}\\ =\frac{1}{4}(G_{jjrr}-G_{m_{1}m_{1}rr}-G_{jjm_{2}m_{2}}+G_{m_{1}m_{1}m_{2}m_{2}})\\ (a_{1j}d_{2rr})=-\frac{\left(\alpha_{1j}\delta_{2rr}\right)}{2}-\frac{\left(\delta_{1jj}\delta_{2rr}\right)}{4}\\ \begin{array}{l} =-\frac{1}{4}(G_{jjrr}-2G_{jjrm_{2}}+G_{jjm_{2}m_{2}})\\ +\frac{1}{4}(G_{m_{1}m_{1}rr}-2G_{m_{1}m_{1}rm_{2}}+G_{m_{1}m_{1}m_{2}m_{2}})\\ (d_{1jj}a_{2r})=-\frac{\left(\delta_{1jj}\alpha_{2r}\right)}{2}-\frac{\left(\delta_{1jj}\delta_{2rr}\right)}{4}\\ \;=-\frac{1}{4}(G_{jjrr}-2G_{jm_{1}rr}+G_{m_{1}m_{1}rr})\\ +\frac{1}{4}(G_{jjm_{2}m_{2}}-2G_{jm_{1}m_{2}m_{2}}+G_{m_{1}m_{1}m_{2}m_{2}})\\ (a_{1j}d_{2rs})=(\alpha_{1j}\delta_{2rs})+\frac{\left(\delta_{1jj}\delta_{2rs}\right)}{2}\\ =\frac{1}{2}(G_{jjrs}-G_{jjrm_{2}}-G_{jjsm_{2}}+G_{jjm_{2}m_{2}})\;\\ \;-\frac{1}{2}(G_{m_{1}m_{1}rs}-G_{m_{1}m_{1}rm_{2}}-G_{m_{1}m_{1}sm_{2}}\\ \;+G_{m_{1}m_{1}m_{2}m_{2}}),\;r<s\\ (d_{1jk}a_{2r})=(\delta_{1jk}\alpha_{2r})+\frac{\left(\delta_{1jj}\delta_{2rr}\right)}{2}\\ =\frac{1}{2}(G_{jkrr}-G_{jm_{1}rr}-G_{km_{1}rr}+G_{m_{1}m_{1}rr})\\ -\frac{1}{2}(G_{jkm_{2}m_{2}}-G_{jm_{1}m_{2}m_{2}}-G_{km_{1}m_{2}m_{2}}\\ +G_{m_{1}m_{1}m_{2}m_{2}}),\;j<k\\ (d_{1jj}d_{2rs})=-\frac{\left(\delta_{1jj}\delta_{2rs}\right)}{2},\;r<s\\ (d_{1jk}d_{2rr})=-\frac{\left(\delta_{1jk}\delta_{2rr}\right)}{2},\;j<k\\ (d_{1jj}d_{2rr})=\frac{\left(\delta_{1jj}\delta_{2rr}\right)}{4}\\ (d_{1jk}d_{2rs})=(\delta_{1jk}\delta_{2rs}),\;j<k,\;r<s \end{array} \end{array}$$

for *j*, *k *= 1, ..., *m*_1 _- 1 and *r*, *s *= 1, ..., *m*_2 _- 1, where the relationships between the parameters of model (14) and model (13) are built based on the equivalency between the two models. The relationships between the parameters of model (14) and the expected genotypic values can then be derived by replacing the parameters of model (13) with the expected genotypic values from the previous established results in (C.1).

**(C.3) **Relationships between parameters of the fully parameterized two-locus model (15) and the expected genotypic values are

$$\left\{\begin{array}{c} \pi_{0}=\mu =G_{m_{1}m_{1}m_{2}m_{2}}\\ \pi_{1j}=\alpha_{1j}=G_{jm_{1}m_{2}m_{2}}-G_{m_{1}m_{1}m_{2}m_{2}}\\ \pi_{2r}=\alpha_{2r}=G_{m_{1}m_{1}rm_{2}}-G_{m_{1}m_{1}m_{2}m_{2}}\\ \eta_{1jj}=2\alpha_{1j}+\delta_{1jj}=G_{jjm_{2}m_{2}}-G_{m_{1}m_{1}m_{2}m_{2}}\\ \eta_{2rr}=2\alpha_{2r}+\delta_{2rr}=G_{m_{1}m_{1}rr}-G_{m_{1}m_{1}m_{2}m_{2}}\\ \eta_{1jk}=\delta_{1jk},j<k;\;\eta_{2rs}=\delta_{2rs},r<s\\ \left(\pi_{1j}\pi_{2r}\right)=\left(\alpha_{1j}\alpha_{2r}\right)=G_{jm_{1}rm_{2}}\\ \;\;\;\;-\left(G_{jm_{1}m_{2}m_{2}}+G_{m_{1}m_{1}rm_{2}}\right)+G_{m_{1}m_{1}m_{2}m_{2}}\\ \left(\pi_{1j}\eta_{2rr}\right)=2\left(\alpha_{1j}\alpha_{2r}\right)+\left(\alpha_{1j}\delta_{2rr}\right)=G_{jm_{1}rr}\\ \;\;\;\;-\left(G_{jm_{1}m_{2}m_{2}}+G_{m_{1}m_{1}rr}\right)+G_{m_{1}m_{1}m_{2}m_{2}}\\ \left(\pi_{1j}\eta_{2rs}\right)=\left(\alpha_{1j}\delta_{2rs}\right),r<s\\ \left(\eta_{1jj}\pi_{2r}\right)=2\left(\alpha_{1j}\alpha_{2r}\right)+\left(\delta_{1jj}\alpha_{2r}\right)=G_{jjrm_{2}}\\ \;\;\;\;-\left(G_{jjm_{2}m_{2}}+G_{m_{1}m_{1}rm_{2}}\right)+G_{m_{1}m_{1}m_{2}m_{2}}\\ \left(\eta_{1jk}\pi_{2r}\right)=\left(\delta_{1jk}\alpha_{2r}\right),j<k\\ \left(\eta_{1jj}\eta_{2rr}\right)=4\left(\alpha_{1j}\alpha_{2r}\right)+2\left(\alpha_{1j}\delta_{2rr}\right)\\ \;\;\;\;\;+2\left(\delta_{1jj}\alpha_{2r}\right)+\left(\delta_{1jj}\delta_{2rr}\right)\\ \;\;\;\;=G_{jjrr}-\left(G_{jjm_{2}m_{2}}+G_{m_{1}m_{1}rr}\right)\\ \;\;\;\;\;+G_{m_{1}m_{1}m_{2}m_{2}}\\ \left(\eta_{1jj}\eta_{2rs}\right)=2\left(\alpha_{1j}\delta_{2rs}\right)+\left(\delta_{1jj}\delta_{2rs}\right)\\ \;\;\;\;=G_{jjrs}-\left(G_{m_{1}m_{1}rs}+G_{jjrm_{2}}+G_{jjsm_{2}}\right)\\ \;\;\;\;\;+\left(G_{m_{1}m_{1}rm_{2}}+G_{m_{1}m_{1}sm_{2}}+G_{jjm_{2}m_{2}}\right)\\ \;\;\;\;\;-G_{m_{1}m_{1}m_{2}m_{2}},r<s\\ \left(\eta_{1jk}\eta_{2rr}\right)=2\left(\delta_{1jk}\alpha_{2r}\right)+\left(\delta_{1jk}\delta_{2rr}\right)\\ \;\;\;\;=G_{jkrr}-\left(G_{jkm_{2}m_{2}}+G_{jm_{1}rr}+G_{km_{1}rr}\right)\\ \;\;\;\;\;+\left(G_{jm_{1}m_{2}m_{2}}+G_{km_{1}m_{2}m_{2}}+G_{m_{1}m_{1}rr}\right)\\ \;\;\;\;\;-G_{m_{1}m_{1}m_{2}m_{2}},j<k\\ \left(\eta_{1jk}\eta_{2rs}\right)=\left(\delta_{1jk}\delta_{2rs}\right),j<k,r<s \end{array}\right)$$

for *j*, *k *= 1, ..., *m*_1 _- 1 and *r*, *s *= 1, ..., *m*_2 _- 1, where the relationships between the parameters of model (15) and model (13) are built based on the equivalency between the two models. The relationships between the parameters of model (15) and the expected genotypic values are then derived by replacing the parameters of model (13) with the expected genotypic values from the previous established results in (C.1).
